# Estimating burden of disease attributable to child maltreatment using findings from the Australian Child Maltreatment Study

**DOI:** 10.1017/S2045796026100572

**Published:** 2026-04-15

**Authors:** Rosana Pacella, Ben Mathews, David Lawrence, Monica Madzoska, Eva Malacova, Holly E. Erskine, Hannah J. Thomas, Daryl J. Higgins, Divna Haslam, Franziska Meinck, James G. Scott

**Affiliations:** 1 Institute for Lifecourse Development, University of Greenwich, London, UK; 2 School of Law, Queensland University of Technology (QUT), Brisbane, QLD, Australia; 3 Australian Centre for Health Law Research, School of Law, Queensland University of Technology (QUT), Brisbane, QLD, Australia; 4 Bloomberg School of Public Health, Johns Hopkins University, Baltimore, MD, USA; 5 School of Population Health, Curtin University, Bentley, WA, Australia; 6 Statistics Unit, QIMR Berghofer, Medical Research Institute, Brisbane, QLD, Australia; 7 Queensland Centre for Mental Health Research, Wacol, QLD, Australia; 8 School of Public Health, The University of Queensland, Brisbane, QLD, Australia; 9 Institute for Health Metrics and Evaluation, University of Washington, Seattle, WA, USA; 10 Child Health Research Centre, The University of Queensland, Brisbane, QLD, Australia; 11 Institute of Child Protection Studies, Australian Catholic University, Melbourne, VIC, Australia; 12 Parenting and Family Support Centre, The University of Queensland, Brisbane, QLD, Australia; 13 School of Social and Political Sciences, University of Edinburgh, Edinburgh, UK; 14 School of Public Health, University of the Witwatersrand, Johannesburg, South Africa; 15 OPTENTIA, Faculty of Humanities, North-West University, Vanderbijlpark, South Africa; 16 Child and Youth Mental Health Service, Children’s Health Queensland Hospital and Health Service, South Brisbane, QLD, Australia

**Keywords:** child abuse, maltreatment, mental health, risk factors, violence

## Abstract

**Aims:**

Prevention of child maltreatment – incorporating physical abuse, sexual abuse, emotional abuse, neglect and exposure to domestic violence – is a clearly defined global policy priority. Global Burden of Disease studies have focused on estimating burden attributable to childhood sexual abuse omitting other forms of child maltreatment. This study aims to estimate burden attributable to child maltreatment using data from the first comprehensive national study, the Australian Child Maltreatment Study (ACMS), accounting for the co-occurrence of multiple forms, the complex impact of multi-type maltreatment and the contribution of interrelated factors.

**Methods:**

We estimated burden attributable to child maltreatment by age and gender for Australia in 2021. Risk–outcome pairs that met criteria for sufficient evidence for a causal relationship were included. Relative risks were estimated as a function of exposure based on data from the ACMS incorporating increased risk with multi-type maltreatment and adjustment for confounding. Levels of exposure in each of the 32 mutually exclusive combinations or patterns of child maltreatment were estimated based on ACMS data by age and gender. The theoretical minimum risk exposure level was determined as no exposure to child maltreatment in the population and population attributable fractions (PAFs) were calculated. Attributable mortality, years of life lost, years lived with disability and disability-adjusted life years (DALYs) were estimated by multiplying PAFs by the relevant burden of disease estimates by age and gender for Australia in 2021. Sensitivity analyses were conducted to assess the robustness of the results. Uncertainty was propagated into attributable burden estimates using Monte Carlo simulation methods.

**Results:**

Overall, child maltreatment accounted for 6.6% (95% uncertainty interval (UI), 6.2–6.9%) of all DALYs for women and 6.4% (95% UI, 6.0–6.7%) of all DALYs for men in Australia in 2021. An estimated 71.2% of self-harm, 57.1% of anxiety disorders and 49.3% of major depressive disorder (MDD) DALYs in women, and 63.8% of self-harm, 55.9% of anxiety disorders and 42.9% of MDD DALYs in men were attributable to child maltreatment.

**Conclusions:**

Child maltreatment contributes to a substantial proportion of burden of disease in Australia, equivalent to leading lifestyle-related risk factors such as high body mass index, high blood pressure and smoking. This research significantly advances knowledge of the disease burden attributable to child maltreatment and provides novel methodology for measuring the impact of all five forms of child maltreatment combined on mental health and health risk behaviours nationally and globally.

## Introduction

Prevention of child maltreatment is a clearly defined global policy priority (UN DESA, 2024). The Australian Child Maltreatment Study (ACMS) provided the first robust national prevalence estimates for all five forms of child maltreatment in Australia – physical abuse, sexual abuse, emotional abuse, neglect and exposure to domestic violence – across childhood (Mathews *et al.*, 2023b). Consistent with other studies, the ACMS found high proportions of multi-type maltreatment with 39.4% (95% CI, 38.1–40.7%) of participants experiencing two or more forms of maltreatment (Higgins *et al.*, 2023). Experiencing multi-type maltreatment was associated with a substantially increased risk of a wide range of mental disorders and health risk behaviours (Lawrence *et al.*, 2023; Scott *et al.*, 2023). Data collected between 2020 and 2021 showed that over two in five Australians had experienced a mental health disorder in their lifetime (Australian Bureau of Statistics (ABS), 2023) with an estimated economic cost of mental illness in Australia of over $200 billion per year (Whiteford, 2022). Given child maltreatment causes mental illness (Norman *et al.*, 2012; Scott *et al.*, 2023; Brauer *et al.*, 2024), it is expected to be a significant risk factor for loss of health in Australia.

Estimating the burden of disease attributable to child maltreatment as a modifiable risk is important for decisions driving international and national priority setting and resource allocation, development and evaluation of effective interventions, comparisons between countries and comparisons with other leading risk factors. However, making accurate estimates of the global burden of child maltreatment has to date been extremely challenging.

International policy on child maltreatment prevention (World Health Organization, 2016, 2020; UN DESA, 2024) and clinical guidance (World Health Organization, 2022) are premised on child maltreatment as a unified phenomenon presenting a population-level risk, with its specific manifestations broadly underpinned by similar social determinants and risk factors at community, familial and individual levels. Given that various types of maltreatment most commonly co-occur and it is less common for an individual to experience only one type of maltreatment (Higgins *et al.*, 2023), assuming independence and merely adding the burden attributable to specific individual sub-types of maltreatment to estimate the overall burden may lead to overestimation while failure to account for the increased risk of adverse health outcomes with multi-type maltreatment may lead to an underestimation of attributable burden. Estimation therefore needs to account for the interdependent effect and interaction of the different maltreatment types as well as their independent effect. Incorporating a method to quantify the effect of child maltreatment as a unified phenomenon rather than as five separate exposures allowing for a nuanced understanding of how different types and patterns of child maltreatment impact health outcomes would provide more accurate estimation of the attributable burden of disease and a clearer guide to prevention efforts.

Furthermore, other factors can distort the relationship between child maltreatment and health outcomes which impacts attributable burden estimation. Initially, published studies and meta-analyses focused on single forms of victimisation may have overestimated the unique association between specific sub-types of child maltreatment and adverse health outcomes, because they did not adequately control for other forms of child maltreatment, other adverse childhood experiences (ACEs) and confounding factors (Finkelhor *et al.*, 2007; Fang *et al.*, 2017). The foundational ACE study, which includes other forms of childhood adversities such as various types of household dysfunction in addition to child maltreatment, established that childhood victimisation, and especially multi-type victimisation, is strongly associated with negative health outcomes across the life course (Felitti *et al.*, 1998) but often ACEs were added to create an ACE score making it difficult to disentangle the effect of child maltreatment from other ACEs.

The Global Burden of Disease (GBD) study provides regular estimates of the proportion of disease burden that can be attributed to particular risk factors. Globally, estimates of disease burden attributable to child maltreatment are limited to estimates for childhood sexual abuse only as a causal risk factor for major depressive disorder (MDD) and alcohol use disorders (AUDs) (Brauer *et al.*, 2024). Despite strong evidence of the causal association between various diseases and non-sexual child maltreatment (Norman *et al.*, 2012), these other forms of child maltreatment are not included in GBD leading to an underestimate of the burden of disease attributable to child maltreatment. This in turn risks an underinvestment in public policy in child safety and child maltreatment prevention.

Two previous studies have attempted quantification of the burden attributable to child maltreatment in Australia as a unified population-health risk rather than simply adding separate exposures, including estimates from the authors prior to ACMS (Moore *et al.*, 2015). In the absence of national prevalence data, this prior study relied on a meta-analysis of regional studies for child maltreatment prevalence and comparability of estimates was compromised by inconsistencies in survey design, samples and research methods (Moore *et al.*, 2015). Moreover, childhood exposure to domestic violence was omitted, so only four forms of child maltreatment were included and only three health outcomes were considered. Furthermore, the study relied on multiple data sources and one small, regional study to estimate co-occurrence of maltreatment types. Despite these limitations, attributable disease burden was still substantial (Moore *et al.*, 2015). The Australian Burden of Disease Study (ABDS) used the population attributable fractions (PAFs) from this previous study by the authors and hence similar data limitations apply. As no data were available to inform trends, the same PAFs were applied to each reference year of the ABDS study. ABDS estimated that child abuse and neglect accounted for 2.5% of all disability-adjusted life years (DALYs) in women and 1.8% of all DALYs in men (Australian Institute of Health and Welfare (AIHW) 2024). A more recent study in Australia estimated similar PAFs, with 21% (95% CI, 13–28%) of depressive disorder and 41% (95% CI, 27–54%) of suicide attempts attributable to child maltreatment (Grummitt *et al.*, 2024). Although this study used prevalence data from ACMS, it included prevalence of any child maltreatment as a dichotomous exposure, excluded exposure to domestic violence in base estimates and did not take multi-type maltreatment into account. In addition, PAFs were calculated using odds ratios derived from a meta-analysis of data from several countries, hence the effect may differ from the Australian population.

There is a pressing need for more comprehensive studies to better understand how maltreatment types interrelate, how multi-type maltreatment influences overall burden of disease and how other factors impact on these complex relationships. However, in addition to methodological challenges, another challenge for estimating attributable burden has been the lack of high-quality data on prevalence of all maltreatment types and on their overlap using operational definitions of known validity to enable improved quantification of the substantial social and economic costs of child maltreatment nationally and globally.

The ACMS is one of the most comprehensive studies on child maltreatment ever conducted, and in addition to prevalence, it also collected data on socio-demographic factors, ACEs and other types of violence against children. Furthermore, important health consequences of child maltreatment were included using standardised measures (Lawrence *et al.*, 2023; Scott *et al.*, 2023), allowing assessment of the increased risk of health risk behaviours and mental health disorders with multi-type maltreatment while controlling for confounding.

The current study uses data from the ACMS and novel methodology to estimate the burden of disease attributable to child maltreatment taking into account: (1) the interrelationships among victimisations in those experiencing multi-type maltreatment; (2) the increased risk of health outcomes and health risk behaviours with multi-type maltreatment; and (3) how other factors including other ACEs affect the relationship.

## Methods

Child maltreatment was treated as a risk factor for loss of health, using counterfactual estimation and comparative risk assessment methods (Jadambaa *et al.*, 2020; Prinsloo *et al.*, 2022; Brauer *et al.*, 2024). This involved comparing the current local health status derived from ACMS data with the theoretical minimum risk exposure level (TMREL) assumed to be zero exposure to child maltreatment. For the purposes of estimating burden of disease attributable to child maltreatment, types and patterns of child maltreatment and the risks associated with different patterns of maltreatment experiences were determined. Below we report details of each step.

### Exposure measures

The ACMS is a cross-sectional survey conducted in Australia in 2021, which collected retrospective self-report data from a nationally representative sample of 8,503 respondents aged 16 years and over (Haslam *et al.*, 2023; Mathews *et al.*, 2023b). In ACMS, child maltreatment was assessed using the Juvenile Victimization Questionnaire (JVQ)-R2: Adapted Version (ACMS). This instrument is based on the well-established JVQ-R2, and its adapted form was the product of a careful process of adaptation and validation reported elsewhere (Mathews *et al.*, 2021, 2023a), with items using more conservative approaches to the nature and chronicity of maltreatment types than many other instruments (Mathews *et al.*, 2020, 2023a). The instrument employed 16 screener items to measure childhood experiences of all five types of child maltreatment (physical abuse, sexual abuse, emotional abuse, neglect and exposure to domestic violence), and specific types of each of these, before the age of 18 years. For physical abuse, we used two items assessing moderate and severe abuse. For sexual abuse, we used four items, with one assessing non-contact abuse, and three assessing different types of contact abuse from touching to completed forced intercourse. For emotional abuse, we used three items assessing verbal hostility/denigration, rejection and emotional unavailability. For neglect, we used three items assessing physical/nutritional neglect, environmental neglect and medical neglect. For exposure to domestic violence, we used four items assessing inter-parental physical violence, serious threats of physical violence, damage to property/pets during arguments, and intimidation or control. Follow-up items captured information on the frequency of the particular maltreatment experience. For physical abuse, sexual abuse and exposure to domestic violence, one or more experience of any screener was counted as a case of that maltreatment type. For emotional abuse, and for neglect, only those experiences occurring over a period of at least weeks were counted. Details of frequency/chronicity of each maltreatment type and screeners are provided elsewhere (Mathews *et al.*, 2023b) and exact wording of the maltreatment items is provided in (Mathews *et al.*, 2023a) (see Supplementary Material Appendix 1 for definitions and details of how the exposures were assessed and Appendix 2 Table A1 for details of the ACMS study population).

### Prevalence of child maltreatment

Weighted estimates of prevalence of child maltreatment in Australia from the ACMS have been previously reported (Mathews *et al.*, 2023b) and are included both as a single exposure and as combinations of multi-type victimisation, with the first age group being 16–19 years and then in 5-year age group categories from 20–24 to 80+ years (Mathews *et al.*, 2023b). In the current study, we derived prevalence for the 32 combinations of the five types of child maltreatment by gender and 5-year age groups. As the ACMS sample of 8,503 participants does not support direct estimation at this level of cross-classification, we used a log-linear model to estimate weighted prevalence (Agresti, 1999). In addition, respondents who identified with a diverse gender (non-binary *n* = 126) were excluded from this analysis, which focused on women (*n* = 4,182) and men (*n* = 4,195) with total *n* = 8,377. The model was fitted using the *CATMOD* procedure in SAS Version 9.4 (SAS Institute Inc., 2013). We modelled the prevalence of the five types of maltreatment and all two-way interactions controlling for age and gender. We adjusted for age, using second order fractional polynomial regression (Royston and Altman, 1994), with the best fitting polynomials being log of age and age squared. The use of log-linear modelling in this context helps smooth out the degree of random error that would exist in direct estimates at this level of disaggregation by applying the constraint of smooth trends by age for each gender using fractional polynomials. Modelled counts and weighted proportions for 32 mutually exclusive patterns of child maltreatment by 5-year age groups and gender are shown in Appendix 3 Table A2.

### Relative risks

Child maltreatment is a risk factor associated with health outcomes, and each combination of risk and outcome is referred to as a risk-outcome pair. We included risk–outcome pairs based on sufficient evidence of a causal relationship (Norman *et al.*, 2012; Lawrence *et al.*, 2023; Scott *et al.*, 2023; Brauer *et al.*, 2024). The next step was to estimate effect size by quantifying the relative risk (RR) of the specified health outcome occurring as a function of exposure to child maltreatment for each risk-outcome pair.

We used ACMS data to calculate RRs for four mental disorders: generalised anxiety disorder (GAD), post-traumatic stress disorder (PTSD), AUDs and MDD, as well as two health risk behaviours: current cigarette smoking (in the past 12 months) and suicide attempts (ever) (answering yes to the question ‘*Have you ever attempted suicide*?’). Diagnoses of GAD (current), PTSD (current), AUD (current) and MDD (lifetime) in ACMS were established using the Mini International Neuropsychiatric Interview (MINI), version 7.0.2 (Lecrubier *et al.*, 1997; Sheehan *et al.*, 1998). For PTSD, the standard MINI module asks if respondents have been exposed to an index trauma and if endorsed, follows by asking if they have ongoing distress or impairment relating to this event. It does not record the index trauma. Importantly the probe questions then determine if the individual meets all of the diagnostic criteria for PTSD. Exposure to trauma is necessary for the diagnosis of PTSD but on its own does not qualify an individual for this diagnosis.

RRs were calculated using log-binomial regression and all models were fitted using the *GLIMMIX* procedure in SAS Version 9.4 (SAS Institute Inc., 2013). We calculated RRs separately for men and for women for all ages combined. We took two different approaches to dealing with the maltreatment patterns in calculating RRs: first considering experiencing the number of types of maltreatment from 0 to 5 (base estimates), and then additionally estimating RRs for the six most common patterns of child maltreatment ((i) exposure to domestic violence, emotional abuse, physical abuse and sexual abuse [EDV + EA + PA + SA]; (ii) exposure to domestic violence, emotional abuse and physical abuse [EDV + EA + PA]; (iii) exposure to domestic violence and emotional abuse [EDV + EA]; (iv) all five types; (v) exposure to domestic violence and physical abuse [EDV + PA] and (vi) exposure to domestic violence and sexual abuse [EDV + SA]) in sensitivity analyses. The other 26 patterns of child maltreatment occurred too infrequently to calculate reliable RR estimates (details of sensitivity analyses in Appendix 4). We ran the models with two different levels of adjustment for other factors – first controlling for age, geographical remoteness and childhood financial stress (simply adjusted model) used in base estimates (Table 1) and sensitivity analysis 1 (Table A3). Then secondly, with additional adjustment for other ACEs (which includes experiencing community violence), as well as peer and sibling bullying victimisation in childhood (fully adjusted model) used in sensitivity analyses 2 and 3 (details presented in Appendix 4, Tables A4-5).

**Table 1.** Relative risks (RRs) of health outcomes by experience of child maltreatment and gender (used in base estimates)

	**Types of child maltreatment**				
**Health outcome**	**1 type**	**2 types**	**3 types**	**4 types**	**5 types**
RR (95% CI)* for men					
MDD	1.7 (1.4–2.1)	2.3 (1.9–2.8)	3.1 (2.5–3.8)	3.4 (2.7–4.4)	3.7 (2.6–5.2)
GAD	1.9 (1.4–2.5)	3.0 (2.2–4.1)	4.2 (3.1–5.6)	6.5 (4.8–8.8)	7.7 (5.2–11.3)
PTSD	2.2 (1.2–3.9)	4.3 (2.4–7.4)	6.5 (3.8–11.3)	14.5 (8.5–24.8)	12.8 (6.4–25.6)
Alcohol use disorders	1.5 (1.3–1.7)	1.5 (1.3–1.7)	1.6 (1.3–1.8)	1.7 (1.4–2.1)	1.6 (1.2–2.3)
Suicide attempt (ever)	2.1 (1.5–3.0)	3.2 (2.3–4.6)	5.8 (4.2–8.1)	8.0 (5.7–11.3)	10.3 (7.0–15.3)
Current smoking	1.5 (1.3–1.8)	1.8 (1.5–2.2)	1.9 (1.6–2.4)	2.4 (1.9–3.0)	2.1 (1.5–3.1)
RR (95% CI)* for women					
MDD	2.0 (1.6–2.4)	2.5 (2.1–3.0)	3.0 (2.5–3.7)	3.3 (2.7–4.0)	3.0 (2.4–3.9)
GAD	1.6 (1.2–2.1)	2.7 (2.1–3.4)	3.1 (2.4–4.0)	4.0 (3.1–5.1)	4.7 (3.6–6.1)
PTSD	2.1 (1.2–3.7)	4.6 (2.7–7.7)	6.0 (3.6–9.9)	12.3 (7.6–19.9)	18.7 (11.4–30.7)
Alcohol use disorders	1.4 (1.2–1.8)	1.9 (1.5–2.3)	2.4 (1.9–2.9)	2.5 (2.0–3.1)	2.5 (1.9–3.3)
Suicide attempt (ever)	2.4 (1.7–3.4)	3.7 (2.6–5.2)	5.4 (3.9–7.5)	8.1 (5.8–11.1)	11.2 (8.0–15.7)
Current smoking	1.5 (1.2–2.0)	1.9 (1.4–2.4)	1.9 (1.5–2.5)	3.0 (2.3–4.0)	3.2 (2.3–4.4)

MDD = major depressive disorder; GAD = generalised anxiety disorder; PTSD = post-traumatic stress disorder.

Reference category is no experience of child maltreatment which is assigned RR = 1.

* Same RRs were assumed to apply across all age groups.

Simply adjusted model (age, childhood financial stress and geographical remoteness).

### Theoretical minimum risk exposure level (TMREL)

The TMREL, or the counterfactual level of risk exposure that minimises disease risk at the population level, was defined as no exposure to child maltreatment in the population (Appendix 5).

### Population attributable fractions

The PAF, or the proportional change in health risk that would occur if exposure to child maltreatment were reduced to the TMREL, was calculated using prevalence estimated for the 32 combinations of the five types of child maltreatment by gender and 5-year age groups, paired with the RRs of disease occurrence given exposure calculated above. The PAFs for this polytomous risk factor for all forms of child maltreatment combined in Australia in 2021 were calculated using customised MS Excel spreadsheets (details described in Appendix 5).

### Attributable burden

Age-gender specific PAFs were then applied to estimates of the burden of disease (deaths, years of life lost (YLLs) due to premature mortality, years lived with disability (YLDs) and DALYs in Australia from GBD 2021 (GBD Collaborative Network, 2021; Ferrari *et al.,* 2024) for the health outcomes in this study to estimate attributable burden (i.e., the proportion of total disease burden attributable to child maltreatment).

In GBD studies, anxiety disorders are aggregated (Baxter *et al.*, 2014), while in ACMS, only PTSD and GAD were measured. PTSD associated with child maltreatment was considered separately and GAD was used as a proxy for the remaining other anxiety disorders. In order to match to GBD disease and injury causes and definitions, we used data from the 2007 National Survey of Mental Health and Wellbeing (Slade *et al.*, 2009) to disaggregate total burden of anxiety disorders in Australia from GBD 2021 into PTSD and other anxiety disorders. The PAF for PTSD was applied to PTSD burden and the PAF for GAD was applied to the remainder, or other anxiety disorders burden of disease estimates, and then added to estimate total anxiety disorders attributable burden.

Self-harm burden estimates for Australia from GBD 2021 include completed suicides and non-fatal injuries from self-harm and suicide attempts. PAFs for suicide attempts (ever) were applied to Australia GBD 2021 burden of disease estimates for intentional self-harm, including deaths (suicides), YLLs (suicides) and YLDs (non-fatal injuries from attempted suicides and self-harm). Attributable DALYs were calculated as the sum of YLLs and YLDs. In the case of current tobacco smoking associated with child maltreatment, the PAFs were applied to the burden attributable to tobacco smoking in Australia quantified in GBD 2021 (GBD Collaborative Network, 2021; Brauer *et al.*, 2024).

### Uncertainty analysis

Monte Carlo simulation modelling techniques were used to present uncertainty ranges around point estimates, reflecting the main sources of sampling uncertainty in the calculations, using Ersatz software version 1.35 (Barendregt, 2017) as an add-in to Excel. For the child maltreatment multiple exposure categories, a Dirichlet distribution (a conjugate of the multinomial distribution) was specified that ensured that the returned random deviates (with binomial distributions) always sum to 1. For the RR input variables, we used the Ersatz random function *ErRelativeRisk* (Barendregt, 2010). For each of the output variables (namely attributable burden as a percentage of total burden in Australia), 95% uncertainty intervals (UIs) were calculated bounded by the 2.5th and 97.5th percentiles of 2,000 iteration values generated.

## Results

### Population attributable fractions

We found a dose–response relationship, whereby those experiencing multi-type maltreatment were at greater risk of developing mental disorders and adopting health risk behaviours compared with those experiencing single-type maltreatment (Table 1). For the base analysis, PAFs for all forms of child maltreatment combined for MDD, PTSD and other anxiety disorders, self-harm, AUDs and tobacco smoking by age and gender are presented in Table 2. As self-reported prevalence for both men and women peaked in the 40–44-year age group and the same RRs were applied across all age groups, PAFs also peaked in the 40–44-year age group. For exposure to all forms of child maltreatment combined (Table 2), the highest PAFs for women were in the age group 40–44 years for PTSD (79.6%), followed by self-harm (73.7%) and other anxiety disorders (56.1%). Similarly, in men, the highest PAFs were in the age group 40–44 years for PTSD (73.5%), followed by intentional self-harm (66.0%) and other anxiety disorders (59.9%). This was a function of the high prevalence in this age group and high RRs for these outcomes particularly with multi-type maltreatment.

**Table 2.** Population attributable fractions (PAFs) for child maltreatment by age group, gender and health outcome (base estimates)

	**Age group (years)**													
**Health outcome**	**15–19^a^**	**20–24**	**25–29**	**30–34**	**35–39**	**40–44**	**45–49**	**50–54**	**55–59**	**60–64**	**65–69**	**70–74**	**75–79**	**80+**
**PAFs for men (%)**														
MDD	44.6	45.0	45.7	46.4	46.8	46.9	46.6	45.9	44.8	43.0	40.8	38.0	34.4	29.4
Other anxiety disorders^&^	57.5	58.1	58.8	59.4	59.9	59.9	59.6	58.8	57.5	55.6	53.1	49.8	45.5	39.3
PTSD	71.3	71.8	72.4	73.0	73.4	73.5	73.1	72.4	71.2	69.4	67.0	63.7	59.3	52.5
Alcohol use disorders	24.3	24.5	24.9	25.3	25.7	25.8	25.7	25.3	24.7	23.8	22.6	21.1	19.3	16.7
Self-harm^$^	63.8	64.3	65.0	65.6	66.0	66.0	65.7	64.9	63.6	61.7	59.3	55.9	51.6	45.1
Current smoking	31.1	31.5	32.1	32.6	33.0	33.1	32.9	32.4	31.5	30.2	28.5	26.3	23.7	20.1
**PAFs for women (%)**														
MDD	51.4	51.7	52.3	52.8	53.2	53.3	53.1	52.6	51.7	50.4	48.7	46.4	43.9	39.8
Other anxiety disorders^&^	54.2	54.6	55.2	55.7	56.0	56.1	55.7	55.1	53.9	52.2	50.1	47.3	44.1	39.0
PTSD	78.4	78.7	79.1	79.5	79.6	79.6	79.2	78.7	77.7	76.3	74.5	71.9	68.9	63.5
Alcohol use disorders	39.2	39.5	40.1	40.6	41.0	41.1	40.8	40.2	39.2	37.7	35.9	33.5	30.8	26.8
Self-harm^$^	72.3	72.6	73.1	73.5	73.7	73.7	73.4	72.8	71.8	70.4	68.5	66.0	63.1	58.0
Current smoking	41.1	41.5	42.1	42.6	42.9	42.9	42.6	42.0	40.9	39.4	37.5	35.1	32.5	28.4

MDD = major depressive disorder; PTSD = post-traumatic stress disorder.

& GAD was used as a proxy for all other anxiety disorders excluding PTSD.

$ PAFs for suicide attempts (ever) were applied to Australia GBD 2021 burden estimates for intentional self-harm (burden from self-inflicted injuries including suicides, non-fatal suicide attempts plus self-harm injuries).

a The 15–19-year age group was used to match GBD age groups although in ACMS this age group consists of 16–19-year-old participants.

Overall, the magnitude of RRs for specific health outcomes was largely driven by number of abuse types experienced rather than specific combinations of maltreatment in sensitivity analyses (see Appendix 4 for detailed comparison of different approaches to estimating RRs). The two different approaches to estimating RRs therefore yielded similar PAFs in the simply adjusted models (Tables 2 and A6). PAFs were lower in the fully adjusted models (Tables A7–8) compared with simply adjusted models. PAFs for child sexual abuse (single exposure and combinations of multi-type victimisation) are presented for comparative purposes (Tables A9–10).

### Attributable burden

Child maltreatment was an important risk factor in Australia, accounting for 6.6% (95% UI, 6.2–6.9%) of all DALYs for women and 6.4% (95% UI, 6.0–6.7%) of all DALYs for men in 2021 (Table 3). The proportions of total disease burden attributable to child maltreatment (across all ages) were higher in women (71.2% of self-harm, 57.1% of anxiety disorders and 49.3% of MDD DALYs) than in men (63.8% of self-harm, 55.9% of anxiety disorders and 42.9% of MDD DALYs) (Table 3). In women, almost half (48.8%) of all mental disorders and health risk behaviours burden estimated in this study was attributable to child maltreatment, compared to 42.2% in men. Total attributable DALYs, however, were higher in men (Table 3).

**Table 3.** Burden of disease attributable to child maltreatment in Australia, 2021 (base estimates)

**Health outcome**	**Proportion of total disease burden attributable to child maltreatment (based on DALYs)**	**Total burden (DALYs)**	**Attributable DALYs**	**Attributable deaths**
**Persons**				
Major depressive disorder	46.7%	208,458	97,359	0
Anxiety disorders^&^	56.7%	191,842	108,714	0
PTSD	69.6%	58,274	40,576	0
Other anxiety disorders	51.0%	133,568	68,138	0
Alcohol use disorders	29.1%	64,852	18,847	115
Self-harm^$^	65.6%	151,212	99,203	2,140
Current smoking	32.3%	345,652	111,500	3,472
**Total burden – mental disorders and health risk behaviours (95% uncertainty interval)**	45.3% (43.4–46.7%)	962,016	435,623 (417,100–449,300)	5,727 (5,200–6,200)
**Total burden – all causes (95% uncertainty interval)**	6.5% (6.2–6.7%)	6,718,079	435,623 (417,100–449,300)	3.3% (3.0–3.5%)
**Women**				
Major depressive disorder	49.3%	123,453	60,912	0
Anxiety disorders^&^	57.1%	118,987	67,982	0
PTSD	72.2%	37,839	27,331	0
Other anxiety disorders	50.1%	81,148	40,652	0
Alcohol use disorders	39.0%	20,347	7,940	38
Self-harm^$^	71.2%	36,469	25,967	560
Current smoking	37.4%	146,257	54,748	1,579
**Total burden – mental disorders and health risk behaviours (95% uncertainty interval)**	48.8% (46.1–51.0%)	445,513	217,550 (205,500–227,400)	2,177 (1,800–2,500)
**Total burden – all causes (95% uncertainty interval)**	6.6% (6.2–6.9%)	3,305,594	217,550 (205,500–227,400)	2.6% (2.2–3.0%)
**Men**				
Major depressive disorder	42.9%	85,006	36,447	0
Anxiety disorders^&^	55.9%	72,856	40,732	0
PTSD	64.8%	20,435	13,246	0
Other anxiety disorders	52.4%	52,421	27,486	0
Alcohol use disorders	24.5%	44,504	10,907	77
Self-harm^$^	63.8%	114,743	73,236	1,580
Current smoking	28.5%	199,395	56,752	1,893
**Total burden – mental disorders and health risk behaviours (95% uncertainty interval)**	42.2% (39.7–44.3%)	516,503	218,074 (205,000–229,000)	3,550 (3,200–3,900)
**Total burden – all causes (95% uncertainty interval)**	6.4% (6.0–6.7%)	3,412,485	218,074 (205,000–229,000)	3.8% (3.4–4.2%)

PTSD = post-traumatic stress disorder.

& GAD in ACMS is used as a proxy for all other anxiety disorders excluding PTSD; PTSD plus other anxiety disorders attributable DALYs were added to estimate total anxiety disorders attributable burden.

$ PAFs for suicide attempts (ever) were applied to Australia GBD 2021 burden estimates for intentional self-harm (burden from self-inflicted injuries including suicides, non-fatal suicide attempts plus self-harm injuries).

Based on simply adjusted model (age, childhood financial stress and geographical remoteness).

The two different approaches for calculating RRs yielded similar attributable burden results (Table 3 and A11 with details described in Appendix 6). Lower estimates of attributable burden were obtained where PAFs were calculated using RRs from fully adjusted models (Tables A12–13) compared with simply adjusted models (Table 3 and Table A11). Burden attributable to child sexual abuse (single exposure and combinations of multi-type victimisation) in Australia, 2021 is presented for comparative purposes in Supplementary Material (Tables A14–15).

## Discussion

This is the first rigorous assessment of the burden of disease attributable to child maltreatment using ACMS data, taking into account the complexities of experiencing different patterns of maltreatment and how these interact to have greater impact. In Australia, child maltreatment accounted for an estimated 6.6% (95% UI, 6.2–6.9%) of all DALYs for women and 6.4% (95% UI, 6.0–6.7%) of all DALYs for men in 2021. The disease burden attributable to child maltreatment is similar to leading lifestyle-related risk factors in Australia (2021), such as high body mass index (7.5% of total DALYs) and smoking and high blood pressure (5.1% of total DALYs) (Brauer *et al.*, 2024).

PAFs were higher in women, and child maltreatment accounted for slightly higher proportions of DALYs in women compared to men. For MDD and anxiety disorders, attributable DALYs were higher in women than in men, but for AUDs, attributable DALYs in men were higher than in women, and for self-harm, attributable DALYs were nearly three times higher in men than in women. Hence, overall, attributable DALYs in men were slightly higher than in women, albeit with overlapping UIs (Table 3).

Our estimates of disease burden attributable to child maltreatment are greater than previously reported in Australia by the authors (Moore *et al.*, 2015). This is explained by some of the previously mentioned limitations, particularly around the use of meta-analytic prevalence estimates of 21.8% and 12.9% for all child maltreatment before the authors published the ACMS with much higher prevalence estimates (67.0% and 59.3%), for women and men, respectively. Furthermore, the inclusion of all five forms of child maltreatment, additional mental health and health risk behaviours outcomes and improved methodology in the current analysis explains the higher estimates.

Although child sexual abuse as a risk factor for loss of health has been quantified in GBD, the contributions of other forms of child maltreatment, the impact of multi-type maltreatment and the aggregation of all forms of child maltreatment combined have been omitted. In GBD 2021, burden attributable to childhood sexual abuse contributed to 0.10% of all global DALYs in 2021 and ranked low compared with other leading risk factors. These results highlight the substantial increase in attributable burden from single exposure to child sexual abuse only, to including all forms of child maltreatment (see Appendix 7). Our study was novel in attempting aggregation while accounting for the co-occurrence of multiple forms of child maltreatment and incorporating the increased risk to health of exposure to multi-type maltreatment. It addresses an important gap, enabling quantification of the overall burden attributable to child maltreatment which is key to inform public health policy and practice.

Nevertheless, the analysis of the relative contribution of child maltreatment to adverse health outcomes remains challenging, given the potential contribution of other factors. Exposure to child maltreatment often co-occurs within the context of other family dysfunction, social deprivation and other environmental stressors that are also associated with mental disorders and health risk behaviours and child maltreatment may also interact with experiences of other forms of violence against children. The selection of confounders in this study was based on existing scientific knowledge and theory and previous work by the ACMS team on factors which can influence both the likelihood of experiencing maltreatment and the risk of developing mental disorders and health risk behaviours as well as interrelated or co-occurring factors which may share the same causal pathway. These included socioeconomic factors (geographical remoteness and childhood financial hardship), family environment and household dysfunction components of ACEs (e.g., parental mental health problems, parental substance use problems, parental separation or divorce), and other childhood experiences of interpersonal violence including community violence component of ACEs and childhood bullying victimisation by peers and siblings.

Australians living in rural and remote areas have shortened life expectancy, poorer health outcomes, higher disease and injury burden and poorer access to and use of health services (Australian Institute of Health and Welfare (AIHW), 2022). Previous analyses of geographical remoteness and childhood financial hardship as potential confounders affecting the strength of the relationship between maltreatment and health found varying degrees of attenuation of these relationships (Lawrence *et al.*, 2023; Pacella *et al.*, 2023; Scott *et al.*, 2023), and hence we adjusted RRs for age, childhood financial stress and geographical remoteness in the base estimates. The richness of the ACMS data also allowed for exploration of other interrelated factors. Family and household dysfunction related ACEs consistently increased risk of multi-type maltreatment (Higgins *et al.*, 2023). Maltreatment increases risk of bullying victimisation and perpetration, and this involvement in bullying independently increases risk of mental disorders (Jadambaa *et al.*, 2020). We investigated additional adjustment for other ACEs and bullying victimisation experiences in sensitivity analyses and found that this further attenuated these complex relationships. For example, the RR for suicide attempts in those experiencing all five types of maltreatment was halved, decreasing from 11.2 (8.0–15.7) and 10.3 (7.0–15.3) (Table 1) in the simply adjusted model (adjusting for age, childhood financial stress and geographical remoteness) to 5.5 (3.8–8.0) and 4.9 (2.3–8.8) (Table A4) after full adjustment for women and men, respectively. Hence the observed association between child maltreatment and health outcomes may have been overestimated in base estimates. Overall, attributable burden still accounted for a substantial proportion of overall DALYs in Australia in 2021 after additionally controlling for other ACEs and bullying victimisation (4.9% [95% UI, 4.6–5.2%], Table A12), which would still place child maltreatment among the leading risk factors in Australia in 2021. This clearly points to the need to fast track efforts to identify, test and disseminate interventions to prevent child maltreatment and reduce disease burden.

### Limitations

ACMS data were collected between April and October 2021 during a period of lockdown due to COVID-19, which may have influenced the results. Indigenous status was included in the survey demographics, and as reported elsewhere (Haslam *et al.*, 2023), the sample included representative participation by Indigenous individuals. However, a limitation of our study is that we did not separately analyse outcomes for this group as our survey was designed as a general population survey and was not designed in accordance with Aboriginal participatory action research principles. In addition, small cell sizes (*n* = 290) precluded meaningful analysis (Appendix 8). The cross-sectional design of the study meant that maltreatment was assessed through retrospective self-report. For many participants, childhood experiences were some decades ago and those respondents with poor health may be more biased towards negative memories. The ACMS was designed to screen for child maltreatment with behaviourally specific items that assessed objective events to maximise the accuracy of participant recall as described in detail elsewhere (Scott *et al.*, 2023; Mathews *et al.*, 2023b). Some of the patterns of child maltreatment occurred too infrequently to calculate reliable RR estimates to pair with the 32 prevalence categories in PAF calculations, but sensitivity analyses showed that the magnitude of RRs for specific health outcomes was largely driven by number of abuse types experienced rather than specific combinations of maltreatment. Models were adjusted for factors known to influence both the likelihood of experiencing maltreatment and the risk of developing mental health problems as well as interrelated factors which may have cumulative detrimental effects on mental health. However, we acknowledge that residual or unmeasured potential confounders may still remain. It is not clear how other ACEs and child maltreatment interact and co-occur throughout childhood and how reducing other adverse family circumstances may reduce the incidence of child maltreatment. In addition, the degree to which these ACEs may be independent risk factors for child maltreatment or each associated in some way with other family and personal characteristics is not well understood. Given the study was cross-sectional, we did not examine the temporality of childhood interpersonal violence experiences. The inclusion of these factors in our models gives a conservative estimate of the impact of child maltreatment on subsequent mental health and health risk behaviours throughout the life course independent of the effects of these other adversities. There may also be important mediators of the effect which are not well understood, where a risk factor may affect another risk factor that lies in the pathway to a disease outcome. For example, high alcohol use is also a risk factor for loss of health which affects some of the same outcomes (namely self-harm/suicide and AUDs) and some of the effect of child maltreatment on these outcomes may be mediated through high alcohol use. In our RR estimation, we did not adjust for mediation as our goal was to capture the direct effect of child maltreatment on outcomes. Child maltreatment significantly increases the risk of experiencing cumulative interpersonal violence victimisation with adverse consequences across the life course. Although we controlled for ACEs including community violence as well as peer and sibling bullying victimisation in sensitivity analyses, some of the effect of child maltreatment on mental disorders and health risk behaviours may be explained by adult victimisation such as intimate partner violence (see Appendix 8). In future studies, it will be interesting to disentangle and better understand these complex causal pathways to estimate the aggregated PAF and burden of disease attributable to various combinations of these psychosocial risk factors.

We also did not model the effects of child maltreatment on co-morbid patterns of mental health disorders which may have overestimated attributable burden. Modelling comorbidity between disorders may reduce the overall burden of disease attributable to child maltreatment but the consistency of burden estimates leaves little doubt that child maltreatment is an important risk factor for mental health issues, and that reducing the incidence of child maltreatment would be expected to contribute to a substantial improvement in population levels of mental health and wellbeing.

PAFs for the first age group (15–19 years) were estimated based on prevalence for 16–19 years and applied to burden of disease estimates for 15–19 years from GBD, but we did not estimate attributable burden for children under 15 years. In addition, respondents with diverse genders were excluded due to small numbers. PAFs for suicide attempts were applied to self-harm burden including fatal burden for completed suicides assuming the same relative risk of death from suicide with experiencing child maltreatment. This was based on research that a previous suicide attempt is highly related to elevated risk of suicide mortality. In addition, PAFs for self-reported suicide attempts were applied to burden of disease estimates meeting International Statistical Classification of Diseases (ICD-10) diagnostic criteria. Another limitation is that our estimate of attributable burden does not include direct deaths from fatal child maltreatment and direct physical injury burden from exposure to non-fatal child maltreatment, as data were not available. Furthermore, although there is suggestive evidence of a link between child maltreatment and physical health outcomes and other health risk behaviours, such as drug use, these were not included but may be considered in future analyses. Hence, the attributable burden may still be an underestimate.

## Conclusions

Child maltreatment contributes to a substantial proportion of burden of disease in Australia, equivalent to leading lifestyle-related risk factors. This research provides novel methodology for measuring the impact of child maltreatment as a unified health risk on mental health and health risk behaviours, accounting for the co-occurrence of maltreatment types and the cumulative impact of multi-type maltreatment experiences. There are therefore significant implications of this research in enabling improved quantification of the overall burden attributable to child maltreatment internationally. This work can form the foundation for future iterations of GBD as improved data become available. Thus, there is also a need to improve the epidemiological data on prevalence and risks through comprehensive national studies such as the ACMS to inform global efforts more accurately.

It is imperative for future national and global studies to incorporate all forms of child maltreatment as risk factors for loss of health to account for the full and extensive burden of child maltreatment enabling governments and policymakers to secure adequate investment in child maltreatment prevention internationally. This will also lead to timely and improved quantification of the substantial lifetime economic costs of child maltreatment. The adverse health effects of child maltreatment are now unequivocally established and given the massive attributable burden and costs of downstream treatment of diseases, urgent investment in effective preventive strategies that reduce child maltreatment is warranted.

## Supporting information

10.1017/S2045796026100572.sm001 Pacella et al. supplementary material Pacella et al. supplementary material

## Data Availability

ACMS information, results and materials are available from the ACMS website https://www.acms.au/for-researchers/. ACMS data have been embargoed for 2 years after conclusion of the official ACMS project and data will be accessible to the general research community after this 2-year embargo period. Under a registered data management plan, final data sets will be stored on the Australian Data Archive, with details for access to be made available on the ACMS website. In order to access the data, researchers will be asked to submit a request outlining their proposed project. These requests will be reviewed by the ACMS team to ensure the proposal is sufficiently covered by the terms of participant consent. MS Excel workbooks for the current analysis for estimates not presented in the main manuscript or additional supplementary tables are available from the authors upon request.

## Supplementary material

The supplementary material for this article can be found at https://doi.org/10.1017/S2045796026100572.
