# Supplementary material for: Estimating burden of disease attributable to child maltreatment using findings from the Australian Child Maltreatment Study
Source: Epidemiol Psychiatr Sci. 2026 Apr 15;35:e25. doi: 10.1017/S2045796026100572 (PMC13122525; doi:10.1017/S2045796026100572)
Supplement: Pacella et al. supplementary material [file S2045796026100572sup001.pdf]

Supplementary Material

Supplement to: Estimating burden of disease attributable to child maltreatment using findings from the Australian Child Maltreatment Study

Table of Contents

Appendix 1 Child maltreatment definitions and assessment of exposure 4
Appendix 2 Details of the study population 6
Appendix 3 Weighted prevalence estimates 8
The six most common patterns of child maltreatment 11
Appendix 4 Relative risks (RRs) 12
Sensitivity analysis 1 12
Sensitivity analysis 2 12
Sensitivity analysis 3 13
Comparing different approaches to estimating RRs 17
Appendix 5 Population attributable fractions 18
Estimating population attributable fractions 18
The theoretical minimum risk exposure level 18
Calculating PAFs for all child maltreatment in base estimates 18
Calculating PAFs for all child maltreatment in sensitivity analyses 19
Calculating PAFs for child sexual abuse 23
Appendix 6 Attributable burden 26
Appendix 7 Child sexual abuse comparison to GBD 2021 33
Appendix 8 Limitations 39
Indigenous status 39
Contribution of multiple risks and mediation 39
Appendix 9 List of abbreviations 40
References 41

List of Tables

Table A1 Demographic characteristics of ACMS respondent sample compared with Australians 16 years and over from the 2016 Australian Census of Population and Housing 6
Table A2 Weighted prevalence estimates for 32 categories of child maltreatment for Australia by age and gender* 8
Table A3 Relative risks (RR) of health outcomes for the six most commonly occurring patterns of child maltreatment for men and women (all ages) (sensitivity analysis 1) 14
Table A4 Relative risks (RR) of health outcomes by experience of child maltreatment for men and women (all ages) with additional adjustment for adverse childhood experiences and bullying victimisation (sensitivity analysis 2) 15
Table A5 Relative risks (RR) of health outcomes for the six most commonly occurring patterns of child maltreatment for men and women with additional adjustment for adverse childhood experiences and bullying victimisation (sensitivity analysis 3) 16
Table A6 Population attributable fractions (PAFs) for all forms of child maltreatment combined, by age group and gender (sensitivity analysis 1) 20
Table A7 Population attributable fractions (PAFs) for all forms of child maltreatment combined, by age group and gender with RRs additionally adjusted for other adverse childhood experiences and bullying victimisation (sensitivity analysis 2) 21
Table A8 Population attributable fractions (PAFs) for all forms of child maltreatment combined, by age group and gender with RRs additionally adjusted for other adverse childhood experiences and bullying victimisation (sensitivity analysis 3) 22
Table A9 Population attributable fractions (PAFs) for child sexual abuse (single exposure only), by age group and gender 24
Table A10 Population attributable fractions (PAFs) for any child sexual abuse (single exposure and combinations of multi-type victimisation where sexual abuse co-occurs with other forms of child maltreatment), by age group and gender 25
Table A11 Burden attributable to child maltreatment in Australia, 2021 (sensitivity analysis 1)* 27
Table A12 Burden attributable to child maltreatment in Australia, 2021 (sensitivity analysis 2)* 29
Table A13 Burden attributable to child maltreatment in Australia, 2021 (sensitivity analysis 3)* 31
Table A14 Burden attributable to child sexual abuse (single exposure only) in Australia, 2021 35
Table A15 Burden attributable to any child sexual abuse (single exposure and combinations of multi-type victimisation) in Australia, 2021 37

Appendix 1 Child maltreatment definitions and assessment of exposure

In ACMS, child maltreatment was assessed with the extensively validated Juvenile Victimization Questionnaire (JVQ)-R2:Adapted Version (Australian Child Maltreatment Study) (Mathews et al. 2021, 2023a). A key strength of the instrument is that it uses robust conceptual models of each of the five maltreatment types, and the translation of these into operational definitions of each maltreatment type in lived experience that are embodied in the survey questions (Mathews et al. 2020), achieving conceptual precision (Hamby 2014), and ensuring accurate measurement of prevalence.

The main exposure measures were physical abuse, sexual abuse, emotional abuse, neglect and exposure to domestic violence before the age of 18 years. Physical abuse was understood as involving intentional acts of physical force against the child by a parent or adult caregiver, excluding lawful corporal punishment. Sexual abuse included contact and non-contact sexual acts by any adult or child, in order to obtain sexual gratification, when the child either lacks the capacity to provide consent or has the capacity but does not provide consent. Emotional abuse included repeated parental behaviours which convey to the child that they are worthless, unloved, unwanted, or only of value when meeting the needs of others. Neglect was the failure by parents to provide the child with the basic necessities of life, as suited to their developmental stage and cultural context. Exposure to domestic violence included seeing or hearing a parent experience assaults, threats, property damage, or coercive control (Mathews et al. 2021).

Participants were asked 16 behaviourally specific questions, which drew out dichotomous responses (Yes/No) about whether the participant had experienced any subdomain of each type of maltreatment (physical abuse: two subdomains; sexual abuse: four; emotional abuse: three; neglect: three; exposure to domestic violence: four). Consistent with best practice, for

emotional abuse, and for neglect, prevalence estimates were calculated using a threshold of requiring exposure over a period of at least weeks in order to satisfy the conceptual model; in contrast, for physical abuse, sexual abuse, and exposure to domestic violence, any experience was counted and included within weighted prevalence estimates (Mathews et al. 2023b).

Appendix 2 Details of the study population

Table A1 Demographic characteristics of ACMS respondent sample compared with Australians 16 years and over from the 2016 Australian Census of Population and Housing

	ACMS sample (2021)			2016 Census*
	Number	Unweighted %	Weighted %	Proportion %
Gender (self-identified)—				
Men	4,195	49.3	48.1	48.8
Women	4,182	49.2	50.9	51.2
Non-binary/other	126	1.5	1.0	-
Total	8,503	100.0	100.0	100.0
Age group—				
16 - 24 years	3,500	41.1	13.6	13.6
25 - 34 years	1,000	11.8	18.2	18.2
35 - 44 years	1,000	11.8	17.0	17.0
45 - 54 years	1,002	11.8	15.7	15.7
55 - 64 years	1,001	11.8	14.5	14.5
65 years or over	1,000	11.8	20.9	20.9
Aboriginal and/or Torres Strait Islander	290	3.4	2.7	2.4
Born in Australia	6,347	74.6	65.9	64.6
Birthplace of parents—				
Both parents born in Australia	4,362	51.3	48.9	45.5
One parent born in Australia	1,351	15.9	10.6	9.8
Both parents born overseas	2,762	32.5	40.2	37.5
Not known	28	0.3	0.3	7.2
Marital status—				
Single/never married	4,046	47.7	29.8	27.4
Living together but not married	918	10.8	11.0	15.3
Married	2,715	32.0	43.9	42.7
Separated/divorced/widowed	803	9.4	15.3	14.6
Highest level of educational attainment—				
Postgraduate degree	1,100	12.9	8.1	8.0
Undergraduate degree	1,859	21.9	17.9	17.7
College certificate/diploma	1,385	16.3	19.6	19.4
Year 12	2,273	26.8	20.6	21.2
Trade certificate	692	8.1	13.5	13.4
Year 10	1,091	12.8	18.3	18.1
Year 9 or below	78	0.9	2.1	2.1
Employment status—				
Employed full-time	3,601	42.5	43.1	39.1
Employed part-time	2,372	27.9	21.3	20.3
Unemployed	724	8.5	7.4	4.5
Not in the labour force	1,779	21.0	28.2	36.0
Residence region—				
Metropolitan	5,798	68.2	64.4	67.0
Regional/rural	2,705	31.8	35.6	33.0
Residence remoteness—				

Major cities	6,247	73.5	69.6	72.1
Inner regional	1,471	17.3	19.1	18.0
Outer regional	658	7.7	9.5	8.1
Remote	83	1.0	1.2	1.0
Very remote	44	0.5	0.6	0.8
SEIFA Index of relative-socio economic advantage and disadvantage—				
Lowest quintile	1,086	12.8	15.6	15.6
2nd quintile	1,180	13.9	15.9	15.9
3rd quintile	1,497	17.6	19.0	19.0
4th quintile	1,938	22.8	20.8	20.8
Highest quintile	2,802	33.0	28.7	28.7
Individual income—				
Less than $500 per week	2,316	27.2	25.1	45.6
$500 - $1249 per week	2,158	25.4	24.0	32.0
$1250 per week or more	2,496	29.4	32.6	22.4
Not stated	1,533	18.0	18.3	-

ACMS= Australian Child Maltreatment Study

SEIFA= Socio-Economic Indexes for Areas. SEIFA ranks geographical areas for relative socio-economic advantage and disadvantage.

*2016 Australian Census data did not include gender options other than male or female.

Adapted from (Haslam et al. 2023).

Appendix 3 Weighted prevalence estimates

Table A2 Weighted prevalence estimates for 32 categories of child maltreatment for Australia by age and gender*

Maltreatment types	Age group (years)													
	15-19[a]	20-24	25-29	30-34	35-39	40-44	45-49	50-54	55-59	60-64	65-69	70-74	75-79	80+	All ages
**Weighted prevalence for men [n (%)]***															
No maltreatment	219 (41.1)	490 (40.4)	108 (39.1)	90 (37.8)	89 (36.8)	86 (36.4)	84 (36.7)	93 (37.6)	97 (39.4)	101 (42.1)	83 (45.4	73 (49.7)	47 (54.8)	48 (61.8)	1708 (40.7)
EDV only	66 (12.3)	150 (12.3)	34 (12.3)	29 (12.1)	28 (11.8)	27 (11.4)	25 (10.9)	26 (10.5)	24 (9.9)	22 (9.3)	16 (8.6)	11 (7.7)	6 (6.7)	4 (5.3)	468 (11.2)
SA only	21 (4.0)	47 (3.9)	11 (3.9)	9 (3.9)	10 (4.1)	10 (4.2)	10 (4.5)	12 (4.8)	13 (5.2)	14 (5.7)	11 (6.3)	10 (6.9)	6 (7.5)	6 (8.1)	191 (4.6)
SA + EDV	7 (1.4)	18 (1.5)	5 (1.7)	5 (1.9)	5 (2.1)	5 (2.2)	5 (2.3)	6 (2.2)	5 (2.2)	5 (2.0)	3 (1.7)	2 (1.4)	1 (1.1)	1 (0.7)	72 (1.7)
PA only	27 (5.1)	64 (5.2)	15 (5.5)	14 (5.8)	15 (6.2)	16 (6.6)	16 (7.1)	19 (7.6)	20 (8.0)	20 (8.5)	16 (8.8)	13 (9.1)	8 (9.0)	6 (8.4)	269 (6.4)
PA + EDV	21 (3.8)	49 (4.0)	12 (4.2)	11 (4.5)	11 (4.7)	11 (4.9)	11 (4.9)	12 (4.9)	12 (4.7)	11 (4.5)	7 (4.1)	5 (3.5)	2 (2.9)	2 (2.1)	177 (4.2)
PA + SA	6 (1.1)	14 (1.2)	4 (1.3)	3 (1.4)	4 (1.5)	4 (1.7)	4 (1.8)	5 (2.0)	5 (2.1)	5 (2.2)	4 (2.3)	3 (2.3)	2 (2.2)	1 (1.9)	65 (1.6)
PA + SA + EDV	5 (1.0)	14 (1.1)	4 (1.4)	4 (1.6)	4 (1.8)	5 (2.0)	5 (2.1)	5 (2.2)	5 (2.0)	4 (1.8)	3 (1.5)	2 (1.2)	1 (0.8)	0 (0.5)	61 (1.5)
NEG only	4 (0.7)	7 (0.6)	1 (0.5)	1 (0.5)	1 (0.4)	1 (0.4)	1 (0.4)	1 (0.4)	1 (0.5)	1 (0.5)	1 (0.6)	1 (0.7)	1 (0.8)	1 (0.9)	23 (0.5)
NEG + EDV	2 (0.4)	5 (0.4)	1 (0.4)	1 (0.3)	1 (0.3)	1 (0.3)	1 (0.3)	1 (0.2)	1 (0.2)	0 (0.2)	0 (0.2)	0 (0.2)	0 (0.1)	0 (0.1)	13 (0.3)
NEG + SA	1 (0.1)	1 (0.1)	0 (0.1)	0 (0.1)	0 (0.1)	0 (0.1)	0 (0.1)	0 (0.1)	0 (0.1)	0 (0.1)	0 (0.1)	0 (0.1)	0 (0.2)	0 (0.2)	4 (0.1)
NEG + SA + EDV	1 (0.1)	1 (0.1)	0 (0.1)	0 (0.1)	0 (0.1)	0 (0.1)	0 (0.1)	0 (0.1)	0 (0.1)	0 (0.1)	0 (0.1)	0 (0.0)	0 (0.0)	0 (0.0)	3 (0.1)
NEG + PA	1 (0.2)	2 (0.2)	0 (0.2)	0 (0.2)	0 (0.2)	1 (0.2)	1 (0.2)	1 (0.2)	1 (0.2)	1 (0.2)	0 (0.2)	0 (0.2)	0 (0.2)	0 (0.1)	8 (0.2)
NEG + PA + EDV	1 (0.3)	3 (0.3)	1 (0.3)	1 (0.3)	1 (0.3)	1 (0.3)	1 (0.3)	1 (0.3)	1 (0.3)	1 (0.2)	0 (0.2)	0 (0.1)	0 (0.1)	0 (0.0)	11 (0.3)
NEG + PA + SA	0 (0.1)	1 (0.1)	0 (0.1)	0 (0.1)	0 (0.1)	0 (0.1)	0 (0.1)	0 (0.1)	0 (0.1)	0 (0.1)	0 (0.1)	0 (0.1)	0 (0.1)	0 (0.0)	3 (0.1)
NEG + PA + SA + EDV	1 (0.1)	2 (0.1)	0 (0.2)	0 (0.2)	0 (0.2)	0 (0.2)	0 (0.2)	0 (0.2)	0 (0.2)	0 (0.1)	0 (0.1)	0 (0.1)	0 (0.0)	0 (0.0)	6 (0.1)
EA only	14 (2.7)	33 (2.7)	8 (2.7)	7 (2.8)	7 (2.8)	7 (2.8)	6 (2.8)	7 (2.7)	6 (2.6)	6 (2.4)	4 (2.2)	3 (1.9)	1 (1.6)	1 (1.2)	109 (2.6)
EA + EDV	30 (5.6)	65 (5.4)	14 (5.1)	11 (4.8)	11 (4.4)	9 (4.0)	8 (3.6)	8 (3.2)	7 (2.8)	6 (2.3)	4 (1.9)	2 (1.6)	1 (1.2)	1 (0.8)	176 (4.2)

EA + SA	2 (0.4)	5 (0.4)	1 (0.4)	1 (0.5)	1 (0.5)	1 (0.5)	1 (0.5)	1 (0.6)	1 (0.6)	1 (0.6)	1 (0.6)	1 (0.5)	0 (0.5)	0 (0.4)	20 (0.5)
EA + SA + EDV	6 (1.1)	13 (1.1)	3 (1.2)	3 (1.2)	3 (1.2)	3 (1.2)	3 (1.2)	3 (1.1)	2 (1.0)	2 (0.9)	1 (0.7)	1 (0.6)	0 (0.4)	0 (0.3)	43 (1.0)
EA + PA	7 (1.3)	18 (1.5)	4 (1.6)	4 (1.8)	5 (2.0)	5 (2.2)	5 (2.3)	6 (2.3)	6 (2.3)	5 (2.2)	4 (2.1)	3 (1.8)	1 (1.5)	1 (1.0)	74 (1.8)
EA + PA + EDV	37 (7.0)	88 (7.2)	21 (7.4)	18 (7.5)	18 (7.5)	17 (7.2)	16 (6.9)	16 (6.4)	14 (5.8)	12 (5.2)	8 (4.4)	5 (3.6)	2 (2.8)	1 (1.9)	274 (6.5)
EA + PA + SA	3 (0.5)	7 (0.6)	2 (0.6)	2 (0.7)	2 (0.8)	2 (0.8)	2 (0.9)	2 (1.0)	2 (1.0)	2 (1.0)	2 (1.0)	1 (0.9)	1 (0.8)	0 (0.6)	30 (0.7)
EA + PA + SA + EDV	16 (3.1)	42 (3.5)	11 (3.9)	10 (4.3)	11 (4.6)	11 (4.7)	11 (4.7)	11 (4.5)	10 (4.1)	9 (3.6)	6 (3.1)	4 (2.4)	2 (1.8)	1 (1.1)	154 (3.7)
EA + NEG	1 (0.2)	2 (0.2)	0 (0.2)	0 (0.1)	0 (0.1)	0 (0.1)	0 (0.1)	0 (0.1)	0 (0.2)	0 (0.2)	0 (0.2)	0 (0.2)	0 (0.3)	0 (0.3)	8 (0.2)
EA + NEG + EDV	6 (1.0)	10 (0.8)	2 (0.7)	1 (0.6)	1 (0.5)	1 (0.4)	1 (0.4)	1 (0.3)	1 (0.3)	1 (0.3)	1 (0.3)	0 (0.3)	0 (0.3)	0 (0.2)	26 (0.6)
EA + NEG + SA	0 (0.1)	1 (0.1)	0 (0.0)	0 (0.0)	0 (0.0)	0 (0.0)	0 (0.0)	0 (0.0)	0 (0.0)	0 (0.1)	0 (0.1)	0 (0.1)	0 (0.1)	0 (0.2)	2 (0.1)
EA + NEG + SA + EDV	2 (0.4)	4 (0.3)	1 (0.3)	1 (0.2)	0 (0.2)	0 (0.2)	0 (0.2)	0 (0.2)	0 (0.2)	0 (0.2)	0 (0.2)	0 (0.2)	0 (0.2)	0 (0.2)	11 (0.3)
EA + NEG + PA	1 (0.2)	3 (0.2)	1 (0.2)	1 (0.2)	1 (0.3)	1 (0.3)	1 (0.3)	1 (0.3)	1 (0.3)	1 (0.4)	1 (0.4)	0 (0.3)	0 (0.3)	0 (0.2)	11 (0.3)
EA + NEG + PA + EDV	13 (2.4)	29 (2.4)	6 (2.3)	5 (2.3)	5 (2.2)	5 (2.1)	5 (2.0)	5 (1.8)	4 (1.7)	4 (1.5)	2 (1.2)	1 (1.0)	1 (0.8)	0 (0.5)	85 (2.0)
EA + NEG + PA + SA	1 (0.2)	2 (0.1)	0 (0.1)	0 (0.1)	0 (0.2)	0 (0.2)	0 (0.2)	0 (0.2)	1 (0.2)	1 (0.2)	0 (0.2)	0 (0.3)	0 (0.3)	0 (0.3)	7 (0.2)
All five types	11 (2.1)	26 (2.1)	6 (2.1)	5 (2.1)	5 (2.1)	5 (2.0)	4 (1.9)	4 (1.8)	4 (1.6)	4 (1.5)	2 (1.3)	2 (1.1)	1 (0.8)	0 (0.6)	79 (1.9)
Child maltreatment	315 (58.9)	724 (59.6)	169 (60.9)	149 (62.2)	152 (63.2)	149 (63.6)	146 (63.3)	154 (62.4)	149 (60.6)	140 (57.9)	100 (54.6)	73 (50.3)	38 (45.2)	29 (38.2)	2487 (59.3)
**Total N**	**534**	**1214**	**277**	**239**	**241**	**235**	**230**	**247**	**246**	**241**	**183**	**146**	**85**	**77**	**4195**

Maltreatment types	Age group (years)													
	**15-19[a]**	**20-24**	**25-29**	**30-34**	**35-39**	**40-44**	**45-49**	**50-54**	**55-59**	**60-64**	**65-69**	**70-74**	**75-79**	**80+**	**All ages**
**Weighted prevalence for women [n (%)]***															
No maltreatment	153 (33.5)	396 (32.9)	68 (31.7)	78 (30.5)	79 (29.5)	772 (29.1)	81 (29.3)	74 (30.2)	77 (31.8)	91 (34.2)	74 (37.3)	65 (41.2)	35 (45.4)	36 (51.9)	1380 (33.0)
EDV only	41 (8.9)	108 (8.9)	19 (8.8)	22 (8.6)	23 (8.4)	20 (8.1)	21 (7.8)	18 (7.4)	17 (7.1)	18 (6.7)	12 (6.3)	9 (5.7)	4 (5.1)	3 (4.2)	335 (8.0)
SA only	34 (7.4)	88 (7.3)	16 (7.2)	19 (7.3)	20 (7.5)	19 (7.8)	23 (8.2)	22 (8.8)	23 (9.7)	28 (10.7)	23 (11.8)	21 (13.0)	11 (14.3)	11 (15.7)	357 (8.5)
SA + EDV	13 (2.9)	38 (3.2)	8 (3.6)	10 (3.9)	12 (4.3)	11 (4.5)	13 (4.7)	11 (4.7)	11 (4.5)	11 (4.1)	7 (3.7)	5 (3.0)	2 (2.4)	1 (1.6)	153 (3.7)
PA only	12 (2.6)	33 (2.7)	6 (2.8)	8 (3.0)	9 (3.2)	8 (3.4)	10 (3.6)	10 (3.9)	10 (4.1)	12 (4.4)	9 (4.6)	8 (4.8)	4 (4.8)	3 (4.6)	141 (3.4)
PA + EDV	7 (1.5)	19 (1.6)	4 (1.7)	4 (1.8)	5 (1.8)	5 (1.9)	5 (1.9)	5 (1.9)	5 (1.9)	5 (1.8)	3 (1.6)	2 (1.4)	1 (1.2)	1 (0.9)	70 (1.7)
PA + SA	5 (1.2)	15 (1.2)	3 (1.3)	4 (1.4)	4 (1.6)	4 (1.7)	5 (1.9)	5 (2.0)	5 (2.2)	6 (2.3)	5 (2.4)	4 (2.4)	2 (2.3)	1 (2.1)	68 (1.6)
PA + SA + EDV	5 (1.0)	14 (1.2)	3 (1.4)	4 (1.6)	5 (1.8)	5 (2.0)	6 (2.1)	5 (2.1)	5 (2.0)	5 (1.8)	3 (1.5)	2 (1.2)	1 (0.9)	0 (0.5)	62 (1.5)
NEG only	2 (0.5)	5 (0.4)	1 (0.3)	1 (0.3)	1 (0.3)	1 (0.3)	1 (0.3)	1 (0.3)	1 (0.3)	1 (0.3)	1 (0.4)	1 (0.4)	0 (0.5)	0 (0.6)	15 (0.4)
NEG + EDV	1 (0.3)	3 (0.3)	1 (0.3)	1 (0.2)	1 (0.2)	0 (0.2)	1 (0.2)	0 (0.2)	0 (0.2)	0 (0.1)	0 (0.1)	0 (0.1)	0 (0.1)	0 (0.1)	9 (0.2)
NEG + SA	1 (0.2)	2 (0.2)	0 (0.2)	0 (0.1)	0 (0.1)	0 (0.1)	0 (0.1)	0 (0.1)	0 (0.2)	0 (0.2)	0 (0.2)	0 (0.3)	0 (0.3)	0 (0.4)	8 (0.2)
NEG + SA + EDV	1 (0.2)	3 (0.2)	0 (0.2)	1 (0.2)	1 (0.2)	0 (0.2)	1 (0.2)	0 (0.2)	0 (0.2)	0 (0.2)	0 (0.1)	0 (0.1)	0 (0.1)	0 (0.1)	8 (0.2)
NEG + PA	0 (0.1)	1 (0.1)	0 (0.1)	0 (0.1)	0 (0.1)	0 (0.1)	0 (0.1)	0 (0.1)	0 (0.1)	0 (0.1)	0 (0.1)	0 (0.1)	0 (0.1)	0 (0.0)	3 (0.1)
NEG + PA + EDV	0 (0.1)	1 (0.1)	0 (0.1)	0 (0.1)	0 (0.1)	0 (0.1)	0 (0.1)	0 (0.1)	0 (0.1)	0 (0.1)	0 (0.1)	0 (0.0)	0 (0.0)	0 (0.0)	4 (0.1)
NEG + PA + SA	0 (0.1)	1 (0.1)	0 (0.1)	0 (0.1)	0 (0.1)	0 (0.1)	0 (0.1)	0 (0.1)	0 (0.1)	0 (0.1)	0 (0.1)	0 (0.1)	0 (0.1)	0 (0.1)	3 (0.1)
NEG + PA + SA + EDV	1 (0.2)	2 (0.2)	0 (0.2)	1 (0.2)	1 (0.2)	1 (0.2)	1 (0.2)	1 (0.2)	0 (0.2)	0 (0.1)	0 (0.1)	0 (0.1)	0 (0.0)	0 (0.0)	7 (0.2)
EA only	16 (3.5)	42 (3.5)	8 (3.5)	9 (3.5)	10 (3.6)	9 (3.6)	10 (3.5)	8 (3.4)	8 (3.3)	8 (3.1)	6 (2.9)	4 (2.6)	2 (2.2)	1 (1.7)	140 (3.3)
EA + EDV	25 (5.4)	63 (5.2)	11 (4.9)	12 (4.6)	11 (4.2)	9 (3.8)	9 (3.4)	7 (3.0)	6 (2.6)	6 (2.3)	4 (1.9)	2 (1.5)	1 (1.2)	1 (0.8)	168 (4.0)
EA + SA	7 (1.4)	17 (1.4)	3 (1.4)	4 (1.5)	4 (1.5)	4 (1.6)	5 (1.7)	4 (1.7)	4 (1.8)	5 (1.8)	4 (1.8)	3 (1.8)	1 (1.7)	1 (1.4)	65 (1.6)
EA + SA + EDV	15 (3.2)	41 (3.4)	8 (3.5)	9 (3.6)	10 (3.7)	9 (3.6)	10 (3.5)	8 (3.3)	7 (3.0)	7 (2.6)	4 (2.2)	3 (1.8)	1 (1.4)	1 (0.9)	132 (3.2)
EA + PA	6 (1.4)	19 (1.5)	4 (1.7)	5 (1.9)	6 (2.1)	6 (2.2)	7 (2.4)	6 (2.4)	6 (2.4)	6 (2.4)	4 (2.2)	3 (2.0)	1 (1.7)	1 (1.2)	79 (1.9)

EA + PA + EDV	22 (4.7)	59 (4.9)	11 (5.0)	13 (5.1)	13 (5.0)	12 (4.8)	13 (4.6)	11 (4.3)	10 (3.9)	9 (3.5)	6 (3.0)	4 (2.5)	2 (2.0)	1 (1.4)	184 (4.4)
EA + PA + SA	5 (1.2)	15 (1.3)	3 (1.4)	4 (1.6)	5 (1.7)	5 (1.9)	6 (2.1)	5 (2.2)	6 (2.3)	6 (2.3)	5 (2.3)	3 (2.1)	2 (1.9)	1 (1.5)	70 (1.7)
EA + PA + SA + EDV	26 (5.8)	79 (6.5)	16 (7.3)	20 (7.9)	23 (8.4)	21 (8.6)	24 (8.6)	20 (8.2)	19 (7.6)	18 (6.8)	11 (5.8)	7 (4.6)	3 (3.6)	2 (2.3)	288 (6.9)
EA + NEG	1 (0.3)	3 (0.3)	0 (0.2)	0 (0.2)	0 (0.2)	0 (0.2)	1 (0.2)	0 (0.2)	1 (0.2)	1 (0.2)	1 (0.3)	1 (0.3)	0 (0.4)	0 (0.4)	10 (0.2)
EA + NEG + EDV	6 (1.3)	13 (1.0)	2 (0.8)	2 (0.7)	2 (0.6)	1 (0.5)	1 (0.5)	1 (0.4)	1 (0.4)	1 (0.4)	1 (0.4)	1 (0.4)	0 (0.3)	0 (0.3)	31 (0.7)
EA + NEG + SA	1 (0.3)	3 (0.2)	0 (0.2)	0 (0.2)	0 (0.1)	0 (0.1)	0 (0.2)	0 (0.2)	0 (0.2)	1 (0.2)	1 (0.3)	1 (0.4)	0 (0.6)	1 (0.9)	10 (0.2)
EA + NEG + SA + EDV	9 (1.9)	19 (1.5)	3 (1.3)	3 (1.1)	3 (1.0)	2 (0.9)	2 (0.8)	2 (0.8)	2 (0.8)	2 (0.8)	2 (0.8)	1 (0.8)	1 (0.8)	1 (0.8)	50 (1.2)
EA + NEG + PA	1 (0.2)	3 (0.2)	1 (0.2)	1 (0.3)	1 (0.3)	1 (0.3)	1 (0.3)	1 (0.4)	1 (0.4)	1 (0.4)	1 (0.4)	1 (0.4)	0 (0.3)	0 (0.3)	12 (0.3)
EA + NEG + PA + EDV	9 (2.0)	24 (2.0)	4 (2.0)	5 (1.9)	5 (1.9)	4 (1.8)	5 (1.7)	4 (1.5)	3 (1.4)	3 (1.2)	2 (1.1)	1 (0.9)	1 (0.7)	0 (0.5)	71 (1.7)
EA + NEG + PA + SA	2 (0.5)	5 (0.4)	1 (0.4)	1 (0.4)	1 (0.4)	1 (0.5)	1 (0.5)	1 (0.6)	1 (0.6)	2 (0.7)	1 (0.7)	1 (0.8)	1 (0.8)	1 (0.8)	22 (0.5)
All five types	27 (6.0)	73 (6.1)	13 (6.1)	15 (6.1)	16 (5.9)	14 (5.7)	15 (5.4)	12 (5.0)	11 (4.6)	11 (4.1)	7 (3.6)	5 (3.1)	2 (2.5)	1 (1.8)	224 (5.4)
Child maltreatment	304 (66.5)	809 (67.1)	147 (68.3)	177 (69.5)	190 (70.5)	175 (70.9)	194 (70.7)	172 (69.8)	166 (68.2)	175 (65.8)	125 (62.7)	93 (58.8)	43 (54.6)	33 (48.1)	2802 (67.0)
**Total N**	**457**	**1205**	**215**	**255**	**269**	**247**	**275**	**246**	**243**	**266**	**199**	**158**	**78**	**69**	**4182**

EA= emotional abuse, PA= physical abuse, SA= sexual abuse, NEG= neglect, EDV= exposure to domestic violence.

[a]The 15-19 year age group was used to match GBD age groups, although in ACMS this age group consists of 16-19 year old participants.

*Prevalence estimates for 32 categories have been smoothed using a log-linear model and may differ slightly from previously published estimates at the aggregate level. Due to rounding, some totals may not correspond with the sum of the separate figures.

The six most common patterns of child maltreatment

i) exposure to domestic violence, emotional abuse, physical abuse, and sexual abuse (EDV+EA+PA+SA);

ii) exposure to domestic violence, emotional abuse and physical abuse (EDV+EA+PA);

iii) exposure to domestic violence and emotional abuse (EDV+EA);

iv) all five types;

v) exposure to domestic violence and physical abuse (EDV+PA); and

vi) exposure to domestic violence, and sexual abuse (EDV+SA)

Appendix 4 Relative risks (RRs)

Sensitivity analysis 1

In a first sensitivity analysis (sensitivity analysis 1), we calculated relative risks (RRs) for the six most commonly occurring patterns of child maltreatment [i) exposure to domestic violence, emotional abuse, physical abuse, and sexual abuse (EDV+EA+PA+SA); ii) exposure to domestic violence, emotional abuse and physical abuse (EDV+EA+PA); iii) exposure to domestic violence and emotional abuse (EDV+EA); iv) (all five types); v) exposure to domestic violence and physical abuse (EDV+PA); vi) exposure to domestic violence and sexual abuse (EDV+SA)], adjusted for age, childhood financial stress [assessed by asking: *How often did your family experience economic hardship such as finding it difficult to provide food, medical care, or other basic necessities*? Participants were classed as experiencing childhood financial hardship if their response was "somewhat" or "very often"], and geographical remoteness [using the Australian Statistical Geography Standard Remoteness Structure (major cities, inner regional, outer regional or remote) (Australian Bureau of Statistics 2021)] (simply adjusted model) (Table A3). Some of the 32 patterns of child maltreatment occurred too infrequently to calculate reliable RR estimates, so for the remaining low-frequency patterns, we used base case analysis RRs (Table 1 in main manuscript) based on the number of types of child maltreatment experienced in that pattern.

Sensitivity analysis 2

In a further sensitivity analysis (sensitivity analysis 2), RRs of disease occurrence in those experiencing 1,2,3,4 or 5 types of maltreatment compared with those not experiencing child maltreatment were adjusted for age, childhood financial stress, geographical remoteness with additional adjustment for other adverse childhood experiences (ACEs) (including yes responses to seven ACEs: *Did your parents or guardians get divorced or separated? Did a parent or guardian die? Did a parent or guardian go to jail? Were you either a victim of*

violence in your neighbourhood, or did you witness violence in your neighbourhood? Did you live with anyone who was mentally ill, suicidal, or severely depressed? Did you live with anyone who had a problem with alcohol or drugs? Were you treated or judged unfairly because of your race or ethnic group?) and peer and sibling bullying experiences (traditional or cyberbullying) in childhood and adolescence including sibling physical victimisation, sibling verbal victimisation, peer physical victimisation, peer verbal victimisation and peer exclusion from activities or through rumours (Thomas et al. 2025) with yes responses to the question: *At the time, did you feel as though you could not defend yourself or make it stop?* (fully adjusted model) (Table A4).

Sensitivity analysis 3

In a third sensitivity analysis (sensitivity analysis 3), we also calculated RRs for the six most commonly occurring patterns of child maltreatment with additional adjustment for other ACEs and peer and sibling bullying experiences (fully adjusted model) (Table A5). For the remaining low-frequency patterns, we used RRs based on the number of types of child maltreatment experienced in that pattern from the fully adjusted model in sensitivity analysis 2, additionally adjusted for other ACEs and bullying victimisation experiences (Table A4).

Table A3 Relative risks (RR) of health outcomes for the six most commonly occurring patterns of child maltreatment for men and women (all ages) (sensitivity analysis 1)

Health outcome	Pattern of child maltreatment					
	EDV + EA + PA + SA	EDV + EA + PA	EDV + EA	All five types	EDV + PA	EDV + SA
**RR (95% CI)* for Men**						
MDD	3.4 (2.6–4.5)	3.1 (2.5–4.0)	2.0 (1.5-2.8)	3.4 (2.5–4.8)	2.5 (1.8–3.3)	2.2 (1.5–3.5)
GAD	6.3 (4.5–8.8)	3.6 (2.5–5.1)	3.1 (2.1–4.7)	6.9 (4.7–10.1)	2.5 (1.6-3.9)	3.1 (1.8-5.5)
PTSD	13.6 (7.7–24.1)	4.1 (2.0–8.1)	4.1 (1.9–8.9)	10.6 (5.3–21.2)	3.5 (1.6–7.9)	4.8 (1.9–12.6)
Alcohol use disorders	1.9 (1.5–2.4)	1.6 (1.3-2.0)	1.6 (1.3–2.0)	1.6 (1.2–2.2)	1.5 (1.2–1.9)	1.4 (1.0–2.0)
Suicide attempts (ever)	9.9 (7.0–14.0)	5.5 (3.8–7.9)	2.9 (1.8–4.9)	9.0 (6.1–13.4)	2.0 (1.1–3.4)	2.9 (1.4–6.0)
Current Smoking	2.6 (2.0–3.4)	1.9 (1.4–2.4)	1.6 (1.2–2.2)	2.0 (1.4–3.0)	1.9 (1.4–2.5)	2.2 (1.5–3.2)
**RR (95% CI)* for Women**						
MDD	3.2 (2.6–3.9)	2.7 (2.1–3.5)	2.8 (2.2–3.6)	2.9 (2.2–3.7)	2.0 (1.3–3.1)	2.8 (2.1–3.6)
GAD	3.8 (2.9–4.9)	2.8 (2.0–3.8)	2.6 (1.9–3.7)	4.3 (3.3–5.6)	2.1 (1.2–3.6)	2.2 (1.4–3.3)
PTSD	12.0 (7.3–19.9)	3.2 (1.5–6.7)	4.5 (2.3–8.8)	16.5 (10.0-27.1)	3.7 (1.3–10.4)	5.1 (2.6–10.3)
Alcohol use disorders	2.6 (2.0–3.2)	1.6 (1.2–2.3)	1.7 (1.2–2.4)	2.4 (1.8–3.2)	1.5 (0.9–2.6)	2.5 (1.9–3.3)
Suicide attempts (ever)	7.3 (5.2–10.2)	4.5 (3.0–6.9)	2.3 (1.4–4.0)	10.0 (7.1–14.0)	2.2 (1.0–5.1)	3.8 (2.3–6.2)
Current smoking	3.1 (2.4–4.2)	1.3 (0.8–2.1)	1.2 (0.7–1.9)	3.0 (2.2–4.1)	1.7 (0.9–3.1)	1.7 (1.1–2.7)

MDD= major depressive disorder; GAD= generalised anxiety disorder; PTSD= post-traumatic stress disorder.

EA= emotional abuse, PA= physical abuse, SA= sexual abuse, NEG= neglect, EDV= exposure to domestic violence.

*Same RRs were assumed to apply across all age groups.

Simply adjusted model (age, childhood financial stress and geographical remoteness).

Table A4 Relative risks (RR) of health outcomes by experience of child maltreatment for men and women (all ages) with additional adjustment for adverse childhood experiences and bullying victimisation (sensitivity analysis 2)

Health outcome	Types of child maltreatment				
	1 type	2 types	3 types	4 types	5 types
**RR (95% CI)* for Men**					
MDD	1.6 (1.3–2.0)	2.0 (1.6–2.5)	2.6 (2.0–3.2)	2.6 (2.0–3.5)	2.6 (1.8–3.8)
GAD	1.6 (1.2–2.2)	1.8 (1.2–2.8)	2.1 (1.5–3.6)	2.4 (1.4–4.2)	3.1 (2.4–5.8)
PTSD	1.7 (0.9–3.1)	2.6 (1.4–4.6)	3.4 (1.9–6.1)	4.4 (2.8–9.7)	4.2 (1.8–8.7)
Alcohol use disorders	1.4 (1.2–1.6)	1.2 (1.1–1.5)	1.3 (1.1–1.5)	1.3 (1.1–.1.6)	1.1 (0.8–1.6)
Suicide-attempt (ever)	1.8 (1.3–2.6)	2.5 (1.8–3.6)	3.3 (2.1–5.1)	3.9 (3.1–7.4)	4.9 (2.3–8.8)
Current smoking	1.4 (1.2–1.7)	1.6 (1.2–2.1)	1.4 (1.1–2.2)	1.4 (1.2–2.1)	1.5 (1.2–2.5)
**RR (95% CI)* for Women**					
MDD	1.9 (1.5–2.4)	2.2 (1.8–2.6)	2.5 (2.0–3.0)	2.5 (2.0–3.2)	2.6 (2.2–3.7)
GAD	1.4 (1.1–1.8)	1.9 (1.5–2.5)	2.3 (1.7–2.9)	2.5 (1.8–3.3)	2.7 (1.9–3.6)
PTSD	1.8 (1.1–3.1)	3.8 (2.3–6.5)	4.1 (2.7–7.7)	6.1 (2.9–10.8)	6.7 (4.2–14.4)
Alcohol use disorders	1.2 (1.0–1.5)	1.5 (1.3–1.9)	1.6 (1.3–2.1)	1.7 (1.3–2.3)	1.8 (1.3–2.4)
Suicide attempts (ever)	2.0 (1.4–2.9)	2.4 (1.6–3.6)	3.7 (2.6–5.2)	4.6 (3.2–6.5)	5.5 (3.8–8.0)
Current smoking	1.4 (1.1–1.8)	1.6 (1.2–2.1)	1.5 (1.1–2.0)	1.9 (1.4–2.7)	1.6 (1.1–2.5)

MDD=major depressive disorder; GAD= generalised anxiety disorder, PTSD= post-traumatic stress disorder.

*Same RRs were assumed to apply across all age groups.

Fully adjusted model [age, childhood financial stress, geographical remoteness, adverse childhood experiences (including community violence) and peer and sibling bullying victimisation experiences].

Table A5 Relative risks (RR) of health outcomes for the six most commonly occurring patterns of child maltreatment for men and women with additional adjustment for adverse childhood experiences and bullying victimisation (sensitivity analysis 3)

Health outcome	Pattern of child maltreatment					
	**EDV + EA + PA + SA**	**EDV + EA + PA**	**EDV + EA**	**All five types**	**EDV + PA**	**EDV + SA**
**RR (95% CI)* for Men**						
MDD	1.9 (1.6–2.5)	1.8 (1.5–2.4)	1.4 (1.1–1.8)	1.9 (1.5–2.3)	1.6 (1.3–2.1)	1.5 (1.1–2.0)
GAD	2.7 (2.2–3.4)	1.9 (1.6–2.3)	1.8 (1.5–2.3)	2.1 (1.8–2.5)	1.6 (1.3–2.2)	2.2 (1.8–2.6)
PTSD	2.1 (1.6–2.9)	1.4 (1.1–1.9)	1.4 (1.1–1.9)	1.8 (1.4–2.3)	1.3 (1.0–1.9)	1.5 (1.2–2.0)
Alcohol use disorders	1.5 (1.2–2.1)	1.3 (1.0–1.7)	1.4 (1.1–1.8)	1.4 (1.1–1.8)	1.2 (0.9–1.5)	1.3 (1.0–1.7)
Suicide attempts (ever)	2.0 (1.4–2.8)	1.5 (1.1–2.1)	1.9 (1.4–2.5)	2.8 (2.1–3.6)	1.4 (1.1–1.9)	1.8 (1.2–2.6)
Current smoking	1.3 (0.9–1.8)	1.3 (0.9–1.8)	1.4 (1.0–1.9)	1.3 (1.0–1.7)	1.4 (1.1–1.8)	1.5 (1.2–1.9)
**RR (95% CI)* for Women**						
MDD	2.0 (1.7–2.4)	1.9 (1.6–2.3)	2.0 (1.7–2.4)	1.7 (1.4–2.1)	1.6 (1.2–2.0)	2.0 (1.6–2.5)
GAD	2.2 (1.8–2.7)	1.8 (1.5–2.2)	2.1 (1.8–2.5)	2.2 (1.8–2.6)	1.6 (1.3–2.2)	1.5 (1.2–2.0)
PTSD	3.1 (1.5–7.7)	1.8 (1.4–2.4)	2.3 (1.8–2.8)	3.2 (1.5–7.8)	1.4 (1.1–2.0)	2.4 (1.9–3.0)
Alcohol use disorders	2.1 (1.5–2.8)	1.5 (1.0–2.1)	1.5 (1.1–2.0)	2.1 (1.5–2.7)	1.3 (0.9–2.1)	1.9 (1.2–2.5)
Suicide attempts (ever)	2.8 (2.2–3.6)	1.7 (1.3–2.5)	1.6 (1.1–2.2)	3.0 (2.2–4.1)	2.1 (1.5–2.8)	2.5 (1.9–3.2)
Current smoking	2.2 (1.8–2.6)	1.1 (0.8–1.4)	1.0 (0.7–1.5)	1.7 (1.2–2.3)	1.6 (1.1–2.2)	1.2 (0.8–1.5)

MDD= major depressive disorder; GAD= generalised anxiety disorder, PTSD= post-traumatic stress disorder.

EA= emotional abuse, PA= physical abuse, SA= sexual abuse, NEG= neglect, EDV= exposure to domestic violence.

*Same RRs were assumed to apply across all age groups.

Fully adjusted model [age, childhood financial stress, geographical remoteness, adverse childhood experiences (including community violence) and peer and sibling bullying victimisation experiences].

Comparing different approaches to estimating RRs

When exploring the six most common combinations of maltreatment in sensitivity analysis, RRs for the combination of having experienced four types of maltreatment (exposure to domestic violence, emotional abuse, physical abuse and sexual abuse) were high and for most health outcomes of similar magnitude to RRs for exposure to all five types of child maltreatment in men (Table A3). For women, however, there was a more pronounced dose response with experiencing all five types of maltreatment yielding higher RRs than 4 types, particularly for suicide attempts and PTSD (Table A3). In men, the different combinations of two types of maltreatment (EDV+PA; EDV+SA; and EDV+EA) yielded similar RRs for most health outcomes. In women, although the combination of two types of maltreatment including sexual abuse (EDV+SA) yielded slightly higher RRs for suicide attempts and PTSD than combinations without sexual abuse (EDV+PA and EDV+EA), experiencing three types of abuse without sexual abuse (EDV + EA + PA) yielded higher RRs than EDV+SA for these suggesting that the magnitude of RRs for specific health outcomes was largely driven by number of abuse types experienced rather than specific combinations of maltreatment. The two different approaches to estimating RRs therefore yielded similar RRs in the simply adjusted models (Table 1 in main manuscript and Table A3). RRs were lower in the fully adjusted models (Tables A4 and A5).

Appendix 5 Population attributable fractions

Estimating population attributable fractions

The population attributable fractions (PAFs) for this polytomous risk factor for all forms of child maltreatment combined in Australia in 2021 were calculated using customised MS Excel spreadsheets using the formula:

$$PAF_{oag} = \frac{\sum_{x=l}^{u} RR_{oag}(x)P_{ag}(x) - RR_{oag}(TMREL_{ag})}{\sum_{x=l}^{u} RR_{oag}(x)P_{ag}(x)}$$

Where PAF_{oag} is the PAF for cause *o* (health outcome) due to child maltreatment as a risk factor for age group *a* and gender *g*; $RR_{oag}(x)$ is the RR as a function of exposure level *x* for cause *o*, age group *a* and gender *g* with exposure levels ranging from *l* to *u*; $P_{ag}(x)$ is the proportion of the population in the risk group (prevalence) for age group *a*, and gender *g*; and $TMREL_{ag}$ is the TMREL for age group *a* and gender *g* (Brauer et al. 2024).

ACMS prevalence for 16-19 years was assumed to apply to the 15-19 years age group in this study to match GBD age groupings, and the same gender specific RRs were assumed to apply across all age groups.

The theoretical minimum risk exposure level

The theoretical minimum risk exposure level (TMREL) or the counterfactual level of risk exposure is the risk exposure that is both theoretically possible and minimises risk in the exposed population. TMREL was defined as no exposure to child maltreatment across all ages and both genders (the counterfactual where the prevalence of child maltreatment is 0% and prevalence of no child maltreatment is 100% and RR=1 for all causes).

Calculating PAFs for all child maltreatment in base estimates

For the base estimates, prevalence estimates for the 32 specified categories of child maltreatment experiences by five-year age group (15-19 to 80+ years) and gender (Table A2) were paired with the RR of the specified health outcome for that risk group (Table 1 in main

manuscript) corresponding to the number of maltreatment types experienced from 0 to 5 (for example, any of the 32 exposure levels with three types of maltreatment were paired with corresponding RR for experiencing 3 types of maltreatment) adjusted for age, childhood financial stress and geographic remoteness (Table 1 in main manuscript). Details for estimating PAFs for sensitivity analyses are presented below.

Calculating PAFs for all child maltreatment in sensitivity analyses

Following the same methodology for the base estimates, for sensitivity analysis 2 the prevalence estimates for the 32 specified categories of child maltreatment experiences by five-year age group and gender (Table A2) were paired with corresponding RRs for experiencing 0,1,2,3,4 or 5 types of maltreatment, adjusted for age, childhood financial stress, geographical remoteness, adverse childhood experiences and bullying victimisation (Table A4) with PAFs for sensitivity analysis 2 presented in Table A7. For sensitivity analyses 1 and 3, prevalence estimates for exposure to the six most common patterns of child maltreatment (Table A2) were paired with the corresponding RRs from the simply adjusted (sensitivity analysis 1; Table A3) or the fully adjusted model (sensitivity analysis 3; Table A5) to calculate PAFs by gender and in 5-year age groups (15-19 to 80+ years). For the remaining 26 categories, prevalence estimates were paired with the corresponding RR of the specified health outcome for that risk group corresponding to the number of maltreatment types experienced from 0 to 5 from the simply adjusted (Table 1 in main manuscript) or fully adjusted model (sensitivity analysis 2; Table A4) to calculate PAFs by gender and in 5-year age groups (15-19 to 80+ years). PAFs for sensitivity analysis 1 and 3 are presented in Tables A6 and A8, respectively.

Table A6 Population attributable fractions (PAFs) for all forms of child maltreatment combined, by age group and gender (sensitivity analysis 1)

Health outcome	Age group (years)													
	15-19[a]	20-24	25-29	30-34	35-39	40-44	45-49	50-54	55-59	60-64	65-69	70-74	75-79	80+
**PAFs for Men**														
Major depressive disorder	44.3%	44.8%	45.5%	46.2%	46.7%	46.8%	46.6%	45.9%	44.8%	43.0%	40.9%	38.0%	34.5%	29.4%
Other anxiety disorders[&]	56.4%	56.9%	57.6%	58.3%	58.7%	58.8%	58.4%	57.7%	56.4%	54.4%	52.0%	48.7%	44.5%	38.5%
PTSD	69.2%	69.7%	70.4%	71.0%	71.5%	71.5%	71.3%	70.6%	69.4%	67.6%	65.2%	61.9%	57.5%	50.9%
Alcohol use disorders	24.9%	25.1%	25.6%	26.0%	26.3%	26.4%	26.3%	25.9%	25.2%	24.2%	23.0%	21.4%	19.5%	16.8%
Self-harm[$]	63.4%	64.0%	64.7%	65.4%	65.9%	65.9%	65.6%	64.8%	63.5%	61.6%	59.0%	55.6%	51.2%	44.7%
Current smoking	31.3%	31.8%	32.4%	33.1%	33.6%	33.8%	33.7%	33.2%	32.3%	30.9%	29.2%	26.9%	24.2%	20.5%
**PAFs for Women**														
Major depressive disorder	51.3%	51.6%	52.2%	52.7%	53.0%	53.1%	52.9%	52.4%	51.5%	50.2%	48.5%	46.3%	43.8%	39.7%
Other anxiety disorders[&]	53.0%	53.3%	53.9%	54.3%	54.7%	54.7%	54.3%	53.7%	52.5%	50.9%	48.8%	46.1%	43.1%	38.1%
PTSD	77.7%	78.0%	78.4%	78.7%	78.9%	78.9%	78.6%	78.0%	77.0%	75.6%	73.8%	71.3%	68.2%	62.8%
Alcohol use disorders	38.0%	38.4%	39.1%	39.7%	40.3%	40.5%	40.3%	39.8%	38.9%	37.4%	35.6%	33.2%	30.6%	26.5%
Self-harm[$]	70.8%	71.2%	71.6%	72.0%	72.3%	72.3%	72.0%	71.5%	70.5%	69.1%	67.3%	64.8%	61.9%	57.0%
Current smoking	38.6%	39.0%	39.7%	40.3%	40.7%	40.9%	40.7%	40.2%	39.2%	37.8%	36.1%	33.8%	31.4%	27.5%

PTSD= post-traumatic stress disorder.

[&]GAD was used as a proxy for all other anxiety disorders excluding PTSD.

[$]PAFs for suicide attempts (ever) were applied to Australia GBD 2021 burden estimates for intentional self-harm (burden from self-inflicted injuries, including suicides, non-fatal suicide attempts plus self-harm injuries).

[a]The 15-19 year age group was used to match GBD age groups, although in ACMS this age group consists of 16-19 year old participants.

Based on simply adjusted model (age, childhood financial stress and geographical remoteness).

Table A7 Population attributable fractions (PAFs) for all forms of child maltreatment combined, by age group and gender with RRs additionally adjusted for other adverse childhood experiences and bullying victimisation (sensitivity analysis 2)

Health outcome	Age group (years)													
	15-19[a]	20-24	25-29	30-34	35-39	40-44	45-49	50-54	55-59	60-64	65-69	70-74	75-79	80+
**PAFs for Men**														
Major Depressive disorder	37.8%	38.2%	38.8%	39.4%	39.9%	40.0%	39.7%	39.1%	38.1%	36.5%	34.6%	32.0%	28.9%	24.6%
Other anxiety disorders[&]	34.2%	34.6%	35.1%	35.7%	36.1%	36.1%	35.9%	35.4%	34.4%	33.0%	31.3%	29.0%	26.3%	22.5%
PTSD	48.8%	49.3%	50.1%	50.8%	51.2%	51.3%	51.0%	50.3%	49.0%	47.2%	44.8%	41.7%	37.7%	32.2%
Alcohol use disorders	15.4%	15.6%	15.8%	16.1%	16.3%	16.4%	16.4%	16.2%	15.9%	15.5%	14.8%	14.0%	13.0%	11.5%
Self-harm[$]	48.4%	48.8%	49.5%	50.2%	50.6%	50.7%	50.4%	49.7%	48.5%	46.7%	44.5%	41.5%	37.8%	32.5%
Current smoking	24.4%	24.7%	25.1%	25.6%	26.0%	26.1%	25.9%	25.5%	24.8%	23.8%	22.4%	20.7%	18.6%	15.8%
**PAFs for Women**														
Major Depressive disorder	45.1%	45.4%	45.9%	46.4%	46.7%	46.9%	46.7%	46.2%	45.4%	44.2%	42.7%	40.7%	38.5%	34.8%
Other anxiety disorders[&]	39.1%	39.5%	40.1%	40.6%	40.9%	41.0%	40.7%	40.1%	39.1%	37.6%	35.8%	33.3%	30.7%	26.7%
PTSD	64.9%	65.3%	65.9%	66.4%	66.7%	66.7%	66.4%	65.8%	64.7%	63.1%	61.1%	58.2%	55.0%	49.5%
Alcohol use disorders	23.9%	24.2%	24.6%	25.0%	25.3%	25.4%	25.2%	24.7%	24.0%	22.8%	21.5%	19.8%	18.0%	15.3%
Self-harm[$]	58.6%	59.0%	59.5%	60.0%	60.3%	60.3%	60.0%	59.3%	58.2%	56.7%	54.7%	52.1%	49.1%	44.3%
Current smoking	27.1%	27.4%	27.8%	28.2%	28.5%	28.6%	28.5%	28.2%	27.5%	26.5%	25.3%	23.8%	22.1%	19.5%

PTSD= post-traumatic stress disorder.

[&]GAD was used as a proxy for all other anxiety disorders excluding PTSD.

[$]PAFs for suicide attempts (ever) were applied to Australia GBD 2021 burden estimates for intentional self-harm (burden from self-inflicted injuries, including suicides, non-fatal suicide attempts plus self-harm injuries).

[a]The 15-19 year age group was used to match GBD age groups, although in ACMS this age group consists of 16-19 year old participants.

Based on fully adjusted model [age, childhood financial stress, geographical remoteness, adverse childhood experiences (including community violence) and peer and sibling bullying victimisation experiences].

Table A8 Population attributable fractions (PAFs) for all forms of child maltreatment combined, by age group and gender with RRs additionally adjusted for other adverse childhood experiences and bullying victimisation (sensitivity analysis 3)

Health outcome	Age group (years)													
	15-19[a]	20-24	25-29	30-34	35-39	40-44	45-49	50-54	55-59	60-64	65-69	70-74	75-79	80+
**PAFs for Men**														
Major depressive disorder	32.2%	32.4%	33.0%	33.5%	34.0%	34.2%	34.2%	33.8%	33.0%	31.8%	30.3%	28.3%	25.8%	22.3%
Other anxiety disorders[&]	33.9%	34.3%	34.9%	35.5%	36.0%	36.1%	35.9%	35.3%	34.4%	33.0%	31.2%	29.0%	26.2%	22.4%
PTSD	38.0%	38.1%	38.6%	39.2%	39.7%	39.9%	39.9%	39.5%	38.7%	37.4%	35.8%	33.6%	30.8%	26.9%
Alcohol use disorders	16.8%	16.9%	17.2%	17.5%	17.7%	17.7%	17.7%	17.4%	17.0%	16.4%	15.7%	14.7%	13.5%	11.8%
Self-harm[$]	40.0%	40.2%	40.6%	41.1%	41.5%	41.7%	41.6%	41.1%	40.2%	38.9%	37.2%	35.0%	32.2%	28.3%
Current smoking	19.2%	19.3%	19.6%	20.0%	20.3%	20.4%	20.4%	20.2%	19.8%	19.2%	18.4%	17.2%	15.8%	13.7%
**PAFs for Women**														
Major depressive disorder	42.5%	42.6%	43.0%	43.4%	43.7%	43.9%	43.7%	43.4%	42.7%	41.7%	40.4%	38.7%	36.7%	33.6%
Other anxiety disorders[&]	37.4%	37.6%	38.0%	38.4%	38.6%	38.6%	38.3%	37.7%	36.8%	35.4%	33.7%	31.5%	29.2%	25.5%
PTSD	58.5%	58.6%	58.9%	59.2%	59.5%	59.5%	59.2%	58.7%	57.8%	56.4%	54.7%	52.3%	49.7%	45.2%
Alcohol use disorders	25.5%	25.9%	26.6%	27.1%	27.6%	27.8%	27.6%	27.1%	26.2%	25.0%	23.4%	21.4%	19.3%	16.1%
Self-harm[$]	53.9%	54.1%	54.5%	54.9%	55.2%	55.3%	55.0%	54.5%	53.6%	52.3%	50.7%	48.4%	45.9%	41.9%
Current smoking	24.6%	25.0%	25.5%	26.1%	26.6%	26.8%	26.9%	26.6%	26.1%	25.2%	24.2%	22.7%	21.2%	18.8%

PTSD= post-traumatic stress disorder.

[&]GAD was used as a proxy for all other anxiety disorders excluding PTSD.

[$]PAFs for suicide attempts (ever) were applied to Australia GBD 2021 burden estimates for intentional self-harm (burden from self-inflicted injuries, including suicides, non-fatal suicide attempts plus self-harm injuries).

[a]The 15-19 year age group was used to match GBD age groups, although in ACMS this age group consists of 16-19 year old participants.

Based on fully adjusted model [age, childhood financial stress, geographical remoteness, adverse childhood experiences (including community violence) and peer and sibling bullying victimisation experiences].

Calculating PAFs for child sexual abuse

PAFs were also estimated for child sexual abuse (single exposure and combinations of multi-type victimisation) for comparative purposes. PAFs for single exposure to 'child sexual abuse only' excluded all other types of child maltreatment in order to compare with GBD 2021 estimates, and 'any child sexual abuse' included all mutually exclusive categories containing sexual abuse (as a polytomous risk factor including single exposure and combinations of multi-type victimisation) (Tables A9-10).

Table A9 Population attributable fractions (PAFs) for child sexual abuse (single exposure only), by age group and gender

Health outcome	Age group (years)													
	15-19[a]	20-24	25-29	30-34	35-39	40-44	45-49	50-54	55-59	60-64	65-69	70-74	75-79	80+
**PAFs for Men**														
Major Depressive disorder	2.7%	2.7%	2.7%	2.7%	2.8%	2.9%	3.0%	3.3%	3.5%	3.8%	4.2%	4.6%	5.0%	5.4%
Other anxiety disorders[&]	3.4%	3.4%	3.4%	3.4%	3.5%	3.7%	3.9%	4.1%	4.5%	4.9%	5.3%	5.8%	6.3%	6.8%
PTSD	4.5%	4.5%	4.5%	4.5%	4.6%	4.8%	5.1%	5.5%	5.9%	6.4%	7.0%	7.6%	8.2%	8.9%
Alcohol use disorders	1.9%	1.9%	1.9%	1.9%	2.0%	2.1%	2.2%	2.3%	2.5%	2.8%	3.0%	3.3%	3.6%	3.9%
Self-harm[$]	4.2%	4.1%	4.1%	4.2%	4.3%	4.5%	4.7%	5.0%	5.4%	5.9%	6.4%	7.0%	7.6%	8.2%
Current smoking	1.9%	1.9%	1.9%	1.9%	2.0%	2.1%	2.2%	2.3%	2.5%	2.8%	3.0%	3.3%	3.6%	3.9%
**PAFs for Women**														
Major Depressive disorder	6.9%	6.8%	6.8%	6.8%	7.0%	7.2%	7.6%	8.1%	8.8%	9.6%	10.5%	11.5%	12.5%	13.6%
Other anxiety disorders[&]	4.3%	4.2%	4.2%	4.2%	4.3%	4.4%	4.7%	5.0%	5.5%	6.0%	6.6%	7.3%	7.9%	8.6%
PTSD	7.5%	7.4%	7.4%	7.4%	7.6%	7.9%	8.3%	8.9%	9.6%	10.5%	11.5%	12.6%	13.6%	14.8%
Alcohol use disorders	2.9%	2.8%	2.8%	2.8%	2.9%	3.0%	3.2%	3.4%	3.7%	4.1%	4.5%	5.0%	5.4%	5.9%
Self-harm[$]	9.4%	9.3%	9.2%	9.3%	9.5%	9.8%	10.3%	11.0%	11.9%	13.0%	14.1%	15.4%	16.6%	18.1%
Current smoking	3.6%	3.5%	3.5%	3.5%	3.6%	3.7%	4.0%	4.2%	4.6%	5.1%	5.6%	6.1%	6.7%	7.3%

PTSD= post-traumatic stress disorder.

[&]GAD was used as a proxy for all other anxiety disorders excluding PTSD.

[$]PAFs for suicide attempts (ever) were applied to Australia GBD 2021 burden estimates for intentional self-harm (burden from self-inflicted injuries, including suicides, non-fatal suicide attempts plus self-harm injuries).

[a]The 15-19 year age group was used to match GBD age groups, although in ACMS this age group consists of 16-19 year old participants.

Based on simply adjusted model (age, childhood financial stress and geographical remoteness).

Table A10 Population attributable fractions (PAFs) for any child sexual abuse (single exposure and combinations of multi-type victimisation where sexual abuse co-occurs with other forms of child maltreatment), by age group and gender

Health outcome	Age group (years)													
	15-19[a]	20-24	25-29	30-34	35-39	40-44	45-49	50-54	55-59	60-64	65-69	70-74	75-79	80+
**PAFs for Men**														
Major depressive disorder	21.5%	22.4%	23.6%	24.7%	25.8%	26.4%	26.6%	26.4%	25.7%	24.5%	22.9%	20.8%	18.5%	15.6%
Other anxiety disorders[&]	34.8%	36.0%	37.6%	39.0%	40.2%	40.8%	40.9%	40.4%	39.3%	37.5%	35.1%	32.1%	28.4%	23.8%
PTSD	51.4%	52.9%	54.6%	56.2%	57.4%	58.1%	58.1%	57.5%	56.2%	54.0%	51.2%	47.4%	42.7%	36.2%
Alcohol use disorders	8.3%	8.7%	9.2%	9.7%	10.2%	10.6%	10.8%	10.8%	10.6%	10.2%	9.7%	9.1%	8.4%	7.5%
Self-harm[$]	41.3%	42.6%	44.2%	45.7%	46.9%	47.6%	47.7%	47.1%	45.9%	43.9%	41.3%	37.9%	33.9%	28.5%
Current smoking	12.7%	13.3%	14.1%	14.9%	15.6%	16.1%	16.3%	16.2%	15.7%	15.0%	14.0%	12.8%	11.5%	9.8%
**PAFs for Women**														
Major depressive disorder	37.2%	37.9%	38.9%	39.9%	40.9%	41.4%	41.5%	41.3%	40.6%	39.5%	38.0%	36.0%	34.0%	30.9%
Other anxiety disorders[&]	42.5%	43.3%	44.3%	45.3%	46.1%	46.4%	46.4%	45.9%	44.8%	43.2%	41.2%	38.6%	35.7%	31.3%
PTSD	72.1%	72.7%	73.5%	74.1%	74.5%	74.6%	74.3%	73.7%	72.6%	71.0%	69.0%	66.1%	62.8%	57.1%
Alcohol use disorders	27.5%	28.2%	29.1%	30.0%	30.8%	31.2%	31.2%	30.9%	30.1%	28.9%	27.4%	25.4%	23.3%	20.3%
Self-harm[$]	63.3%	64.0%	64.9%	65.7%	66.2%	66.5%	66.3%	65.7%	64.7%	63.1%	61.0%	58.3%	55.2%	50.2%
Current smoking	30.6%	31.3%	32.1%	32.9%	33.6%	33.9%	33.8%	33.3%	32.4%	31.1%	29.5%	27.4%	25.2%	22.0%

PTSD= post-traumatic stress disorder.

[&]GAD was used as a proxy for all other anxiety disorders excluding PTSD.

[$]PAFs for suicide attempts (ever) were applied to Australia GBD 2021 burden estimates for intentional self-harm (burden from self-inflicted injuries, including suicides, non-fatal suicide attempts plus self-harm injuries).

[a]The 15-19 year age group was used to match GBD age groups, although in ACMS this age group consists of 16-19 year old participants.

Based on simply adjusted model (age, childhood financial stress and geographical remoteness).

Appendix 6 Attributable burden

The two different approaches for calculating RRs yielded similar attributable burden results. In sensitivity analysis 1, using the approach where PAFs were calculated using RRs based on experiencing the 6 most common patterns of child maltreatment and adjustment for age, childhood financial stress and geographical remoteness, estimates for women [6.5% (95% UI 6.1-6.7%)] and for men [6.4% (95% UI 6.0-6.7%)] (Table A11) were similar to base estimates [using the approach considering experiencing number of types of maltreatment (from 0 to 5) and simply adjusted model] (Table 3 in main manuscript). Again, in sensitivity analysis 2 where PAFs were calculated using RRs for experiencing number of types of maltreatment with adjustment for age, childhood financial stress, geographical remoteness, other ACEs and bullying victimisation (Table A12), estimates were similar to those in sensitivity analysis 3 [4.8% (95% UI 4.4-5.1%) for women and 4.1% (95% UI 3.6-4.5%) for men] with similar adjustment (Table A13).

Lower estimates of attributable burden were obtained where PAFs were calculated using RRs from fully adjusted models (Tables A12-13) compared with simply adjusted models (Table A11 and Table 3 in main manuscript). In sensitivity analysis 2, where RRs for experiencing number of types of maltreatment were additionally adjusted for other ACEs and bullying victimisation experiences, estimates were reduced by about 25% [5.1% (95% UI 4.6-5.5%) or 168,545 (95% UI 153,400-180,600) of all DALYs in women and 4.8% (95% UI 4.3-5.2%) or 163,979 DALYs (147,500-178,300) of all DALYs in men attributable to all forms of child maltreatment (Table A12)] compared with the base estimates [6.6% (95% UI 6.2-6.9%) or 217,550 (205,500-227,400) attributable DALYs in women and 6.4% (95% UI 6.0-6.7%) or 218,074 (205,000-229,000) attributable DALYs in men controlling for age, childhood financial stress and geographical remoteness) (Table 3 in main manuscript)].

Table A11 Burden attributable to child maltreatment in Australia, 2021 (sensitivity analysis 1)*

Health outcome	Proportion of total disease burden attributable to child maltreatment (Based on DALYs)	Total burden (DALYs)	Attributable DALYs	Attributable Deaths
**Persons**				
Major depressive disorder	46.6%	208,458	97,081	0
Anxiety disorders[&]	55.6%	191,842	106,577	0
PTSD	68.6%	58,274	39,970	0
Other anxiety disorders	49.9%	133,568	66,608	0
Alcohol use disorders	29.2%	64,852	18,952	116
Self-harm[$]	65.1%	151,212	98,475	2,124
Current smoking	32.0%	345,652	110,537	3,456
**Total burden - mental disorders and health risk behaviours (95% Uncertainty Interval)**	44.9% (43.1-46.3%)	962,016	431,622 (415,100-445,000)	5,695 (5,200-6,100)
**Total burden all causes (95% Uncertainty Interval)**	6.4% (6.2-6.6%)	6,718,079	431,622 (415,100-445,000)	3.3% (3.0-3.5%)
**Women**				
Major depressive disorder	49.2%	123,453	60,750	0
Anxiety disorders[&]	56.1%	118,987	66,752	0
PTSD	71.6%	37,839	27,084	0
Other anxiety disorders	48.9%	81,148	39,668	0
Alcohol use disorders	38.3%	20,347	7,801	37
Self-harm[$]	69.8%	36,469	25,467	549
Current smoking	35.9%	146,257	52,537	1,521
**Total burden - mental disorders and health risk behaviours (95% Uncertainty Interval)**	47.9% (45.4-50.0%)	445,513	213,308 (202,200-222,700)	2,108 (1,800-2,400)
**Total burden all causes (95% Uncertainty Interval)**	6.5% (6.1-6.7%)	3,305,594	213,308 (202,200-222,700)	2.6% (2.2-2.9%)
**Men**				
Major depressive disorder	42.7%	85,006	36,331	0
Anxiety disorders[&]	54.7%	72,856	39,826	0
PTSD	63.1%	20,435	12,886	0
Other anxiety disorders	51.4%	52,421	26,940	0

Alcohol use disorders	25.1%	44,504	11,151	79
Self-harm[$]	63.6%	114,743	73,008	1,575
Current smoking	29.1%	199,395	57,999	1,934
**Total burden - mental disorders and health risk behaviours (95% Uncertainty Interval)**	42.3% (39.9-44.2%)	516,503	218,315 (206,300-228,300)	3,587 (3,200-3,900)
**Total burden all causes (95% Uncertainty Interval)**	6.4% (6.0-6.7%)	3,412,485	218,315 (206,300-228,300)	3.9% (3.5-4.2%)

PTSD= post-traumatic stress disorder.

[&]GAD in ACMS is used as a proxy for all other anxiety disorders excluding PTSD; PTSD plus other anxiety disorders attributable DALYs were added to estimate total anxiety disorders attributable burden.

[$]PAFs for suicide attempts (ever) were applied to Australia GBD 2021 burden estimates for intentional self-harm (burden from self-inflicted injuries, including suicides, non-fatal suicide attempts plus self-harm injuries).

*In sensitivity analysis 1, RRs were calculated for the six most commonly occurring patterns of child maltreatment.

Based on simply adjusted model (age, childhood financial stress and geographical remoteness).

Table A12 Burden attributable to child maltreatment in Australia, 2021 (sensitivity analysis 2)*

Health outcome	Proportion of total disease burden attributable to child maltreatment (Based on DALYs)	Total burden (DALYs)	Attributable DALYs	Attributable Deaths
**Persons**				
Major depressive disorder	40.5%	208,458	84,447	0
Anxiety disorders[&]	40.5%	191,842	77,753	0
PTSD	54.6%	58,274	31,844	0
Other anxiety disorders	34.4%	133,568	45,909	0
Alcohol use disorders	18.3%	64,852	11,851	73
Self-harm[$]	50.9%	151,212	76,921	1,654
Current smoking	23.6%	345,652	81,553	2,560
**Total burden - mental disorders and health risk behaviours (95% Uncertainty Interval)**	34.6% (32.1-36.4%)	962,016	332,525 (308,900-350,400)	4,287 (3,700-4,800)
**Total burden all causes (95% Uncertainty Interval)**	4.9% (4.6-5.2%)	6,718,079	332,525 (308,900-350,400)	2.4% (2.1-2.8%)
**Women**				
Major depressive disorder	43.3%	123,453	53,491	0
Anxiety disorders[&]	43.9%	118,987	52,196	0
PTSD	60.0%	37,839	22,720	0
Other anxiety disorders	36.3%	81,148	29,476	0
Alcohol use disorders	23.9%	20,347	4,859	23
Self-harm[$]	57.8%	36,469	21,096	453
Current smoking	25.2%	146,257	36,903	1,071
**Total burden - mental disorders and health risk behaviours (95% Uncertainty Interval)**	37.8% (34.4-40.5%)	445,513	168,545 (153,400-180,600)	1,547 (1,200-1,900)
**Total burden all causes (95% Uncertainty Interval)**	5.1% (4.6-5.5%)	3,305,594	168,545 (153,400-180,600)	1.9% (1.4-2.3%)
**Men**				
Major depressive disorder	36.4%	85,006	30,957	0
Anxiety disorders[&]	35.1%	72,856	25,556	0
PTSD	44.6%	20,435	9,124	0
Other anxiety disorders	31.3%	52,421	16,433	0

Alcohol use disorders	15.7%	44,504	6,991	50
Self-harm[$]	48.7%	114,743	55,825	1,200
Current smoking	22.4%	199,395	44,650	1,489
**Total burden - mental disorders and health risk behaviours (95% Uncertainty Interval)**	31.7% (28.6-34.5%)	516,503	163,979 (147,500-178,300)	2,739 (2,300-3,200)
**Total burden all causes (95% Uncertainty Interval)**	4.8% (4.3-5.2%)	3,412,485	163,979 (147,500-178,300)	3.0% (2.5-3.4%)

PTSD= post-traumatic stress disorder.

[&]GAD in ACMS is used as a proxy for all other anxiety disorders excluding PTSD; PTSD plus other anxiety disorders attributable DALYs were added to estimate total anxiety disorders attributable burden.

[$]PAFs for suicide attempts (ever) were applied to Australia GBD 2021 burden estimates for intentional self-harm (burden from self-inflicted injuries, including suicides, non-fatal suicide attempts plus self-harm injuries).

*In sensitivity analysis 2, relative risks were calculated for experiencing 1,2,3,4 or 5 types of child maltreatment versus no maltreatment (zero maltreatment).

Based on fully adjusted model [age, childhood financial stress, geographical remoteness, adverse childhood experiences (including community violence) and peer and sibling bullying victimisation experiences].

Table A13 Burden attributable to child maltreatment in Australia, 2021 (sensitivity analysis 3)*

Health outcome	Proportion of total disease burden attributable to child maltreatment (Based on DALYs)	Total burden (DALYs)	Attributable DALYs	Attributable Deaths
**Persons**				
Major depressive disorder	36.9%	208,458	76,839	0
Anxiety disorders[&]	37.4%	191,842	71,671	0
PTSD	47.1%	58,274	27,441	0
Other anxiety disorders	33.1%	133,568	44,230	0
Alcohol use disorders	19.7%	64,852	12,806	78
Self-harm[$]	43.3%	151,212	65,424	1,411
Current smoking	20.7%	345,652	71,391	2,258
**Total burden - mental disorders and health risk behaviours (95% Uncertainty Interval)**	31.0% (28.8-32.7%)	962,016	298,132 (277,200-314,500)	3,747 (3,200-4,300)
**Total burden all causes (95% Uncertainty Interval)**	4.4% (4.1-4.7%)	6,718,079	298,132 (277,200-314,500)	2.1% (1.8-2.4%)
**Women**				
Major depressive disorder	40.7%	123,453	50,250	0
Anxiety disorders[&]	40.5%	118,987	48,175	0
PTSD	53.7%	37,839	20,320	0
Other anxiety disorders	34.3%	81,148	27,855	0
Alcohol use disorders	25.9%	20,347	5,277	25
Self-harm[$]	53.1%	36,469	19,377	417
Current smoking	24.0%	146,257	35,051	1,025
**Total burden - mental disorders and health risk behaviours (95% Uncertainty Interval)**	35.5% (32.6-38.1%)	445,513	158,130 (145,400-169,800)	1,467 (1,100-1,800)
**Total burden all causes (95% Uncertainty Interval)**	4.8% (4.4-5.1%)	3,305,594	158,130 (145,400-169,800)	1.8% (1.4-2.2%)
**Men**				
Major depressive disorder	31.3%	85,006	26,589	0
Anxiety disorders[&]	32.3%	72,856	23,496	0
PTSD	34.8%	20,435	7,121	0
Other anxiety disorders	31.2%	52,421	16,375	0

Alcohol use disorders	16.9%	44,504	7,529	53
Self-harm[$]	40.1%	114,743	46,047	994
Current smoking	18.2%	199,395	36,341	1,233
**Total burden - mental disorders and health risk behaviours (95% Uncertainty Interval)**	27.1% (24.0-29.7%)	516,503	140,002 (124,200-153,300)	2,280 (1,800-2,700)
**Total burden all causes (95% Uncertainty Interval)**	4.1% (3.6-4.5%)	3,412,485	140,002 (124,200-153,300)	2.5% (2.0-2.9%)

PTSD= post-traumatic stress disorder.

[&]GAD in ACMS is used as a proxy for all other anxiety disorders excluding PTSD; PTSD plus other anxiety disorders attributable DALYs were added to estimate total anxiety disorders attributable burden.

[$]PAFs for suicide attempts (ever) were applied to Australia GBD 2021 burden estimates for intentional self-harm (burden from self-inflicted injuries, including suicides, non-fatal suicide attempts plus self-harm injuries).

*In sensitivity analysis 3, RRs were calculated for the six most commonly occurring patterns of child maltreatment.

Based on fully adjusted model [age, childhood financial stress, geographical remoteness, adverse childhood experiences (including community violence) and peer and sibling bullying victimisation experiences].

Appendix 7 Child sexual abuse comparison to GBD 2021

In the Global Burden of Disease 2021 study (GBD 2021), only major depressive disorder and alcohol use disorders were included as health outcomes for childhood sexual abuse using criteria for convincing or probable evidence. We considered that the evidence for a causal relationship was robust to warrant the inclusion of other health outcomes in this analysis. In GBD 2021, RRs for major depressive disorder (RR=1.56 (95% CI 1.30-1.86) and alcohol use disorders (RR= 2.21 (95% CI (1.15-4.04)) were based on meta-analyses of international studies (Brauer et al. 2024) and used across all countries and applied to both genders and across all age groups. Our analysis derived RRs from ACMS by gender and included the increased risk with multi-type maltreatment for combinations of sexual abuse with other forms of maltreatment.

In GBD 2021, childhood sexual abuse accounted for 0.3% (0.2-0.5%) and 0.1% (0.1-0.2%) of total DALYs in women and men in Australia in 2021 (GBD Collaborative Network 2021, Brauer et al. 2024) which is the same as our estimate for child sexual abuse (single exposure only) when the same two health outcomes are included [0.3% (0.2-0.4%) and 0.1% (0.07-0.15%)] but lower than our estimate when additional health outcomes are included [0.9% (0.6-1.1%) and 0.5% (0.4-0.7%)] of total DALYs in women and men in Australia in 2021 (Table A14). As expected, our estimates of burden attributable to any sexual abuse (as a polytomous risk factor including single exposure and combinations of multi-type victimisation with the increased risk of health outcomes with multi-type maltreatment) were higher than single exposure only [5.4% (5.0-5.6%) and 3.8% (3.5-4.1%) of total DALYs in women and men in Australia in 2021] (Table A15).

Although difficult to make comparisons to GBD 2021, these results highlight the substantial increase in attributable burden from single exposure to child sexual abuse only, to including all forms of child maltreatment (Table 3 in main manuscript). The GBD study's iterative

nature creates significant challenges for comparing results across different updates, as each new iteration supersedes previous ones with revised estimates, updated methodologies, and improved data sources and the entire time series is re-estimated with each new GBD study and hence it is important to note that these comparisons were to GBD 2021 and GBD results may change in future iterations.

Table A14 Burden attributable to child sexual abuse (single exposure only) in Australia, 2021

Health outcome	Proportion of total disease burden attributable to child maltreatment (Based on DALYs)	Total burden (DALYs)	Attributable DALYs	Attributable Deaths
**Persons**				
Major depressive disorder	5.7%	208,458	11,823	0
Anxiety disorders[&]	5.0%	191,842	9,499	0
PTSD	6.8%	58,274	3,964	0
Other anxiety disorders	4.1%	133,568	5,535	0
Alcohol use disorders	2.6%	64,852	1,700	13
Self-harm[$]	6.0%	151,212	9,047	216
Current smoking	3.9%	345,652	13,634	526
**Total burden - mental disorders and health risk behaviours (95% Uncertainty Interval)**	4.8% (3.7-5.7%)	962,016	45,702 (35,800-54,900)	755 (500-1,000)
**Total burden all causes (95% Uncertainty Interval)**	0.7% (0.5-0.8%)	6,718,079	45,702 (35,800-54,900)	0.4% (0.3-0.6%)
**Total burden 2 health outcomes (AUD and MDD) (95% Uncertainty Interval)**	0.2% (0.1-0.3%)	6,718,079	13,522 (9,600-17,400)	0.01% (0.005-0.011%)
**Women**				
Major depressive disorder	7.5%	123,453	9,246	0
Anxiety disorders[&]	5.6%	118,987	6,671	0
PTSD	7.9%	37,839	3,006	0
Other anxiety disorders	4.5%	81,148	3,665	0
Alcohol use disorders	3.3%	20,347	677	4
Self-harm[$]	10.3%	36,469	3,749	89
Current smoking	5.3%	146,257	7,818	291
**Total burden - mental disorders and health risk behaviours (95% Uncertainty Interval)**	6.3% (4.4-8.1%)	445,513	28,160 (19,800-36,000)	384 (200-600)
**Total burden all causes (95% Uncertainty Interval)**	0.9% (0.6-1.1%)	3,305,594	28,160 (19,800-36,000)	0.5% (0.2-0.8%)

**Total burden 2 health outcomes (AUD and MDD) (95% Uncertainty Interval)**	0.3% (0.2-0.4%)	3,305,594	9,922 (6,300-13,500)	0.01% (0.002-0.009%)
**Men**				
Major depressive disorder	3.0%	85,006	2,577	0
Anxiety disorders[&]	3.9%	72,856	2,828	0
PTSD	4.7%	20,435	959	0
Other anxiety disorders	3.6%	52,421	1,869	0
Alcohol use disorders	2.3%	44,504	1,023	9
Self-harm[$]	4.6%	114,743	5,298	127
Current smoking	2.9%	199,395	5,816	235
**Total burden - mental disorders and health risk behaviours (95% Uncertainty Interval)**	3.4% (2.5-4.4%)	516,503	17,542 (12,800-22,800)	371 (200-500)
**Total burden all causes (95% Uncertainty Interval)**	0.5% (0.4-0.7%)	3,412,485	17,542 (12,800-22,800)	0.4% (0.2-0.6%)
**Total burden 2 health outcomes (AUD and MDD) (95% Uncertainty Interval)**	0.1% (0.07-0.15%)	3,412,485	3,600 (2,300-5,000)	0.01% (0.006-0.015%)

PTSD= post-traumatic stress disorder.

[&]GAD in ACMS is used as a proxy for all other anxiety disorders excluding PTSD; PTSD plus other anxiety disorders attributable DALYs were added to estimate total anxiety disorders attributable burden.

[$]PAFs for suicide attempts (ever) were applied to Australia GBD 2021 burden estimates for intentional self-harm (burden from self-inflicted injuries, including suicides, non-fatal suicide attempts plus self-harm injuries).

Based on simply adjusted model (age, childhood financial stress and geographical remoteness).

Table A15 Burden attributable to any child sexual abuse (single exposure and combinations of multi-type victimisation) in Australia, 2021

Health outcome	Proportion of total disease burden attributable to child maltreatment (Based on DALYs)	Total burden (DALYs)	Attributable DALYs	Attributable Deaths
**Persons**				
Major depressive disorder	31.7%	208,458	66,060	0
Anxiety disorders[&]	45.2%	191,842	86,789	0
PTSD	61.1%	58,274	35,582	0
Other anxiety disorders	38.3%	133,568	51,207	0
Alcohol use disorders	15.9%	64,852	10,334	61
Self-harm[$]	49.1%	151,212	74,179	1,602
Current smoking	20.5%	345,652	70,878	2,163
**Total burden - mental disorders and health risk behaviours (95% Uncertainty Interval)**	32.0% (30.4-33.3%)	962,016	308,240 (292,600-320,300)	3,827 (3,400-4,200)
**Total burden all causes (95% Uncertainty Interval)**	4.6% (4.4-4.8%)	6,718,079	308,240 (292,600-320,300)	2.2% (2.0-2.4%)
**Women**				
Major depressive disorder	37.5%	123,453	46,337	0
Anxiety disorders[&]	49.2%	118,987	58,590	0
PTSD	67.2%	37,839	25,427	0
Other anxiety disorders	40.9%	81,148	33,163	0
Alcohol use disorders	29.2%	20,347	5,935	29
Self-harm[$]	63.6%	36,469	23,182	500
Current smoking	29.4%	146,257	42,988	1,234
**Total burden - mental disorders and health risk behaviours (95% Uncertainty Interval)**	39.7% (37.3-41.8%)	445,513	177,032 (166,300-186,100)	1,762 (1,500-2,000)
**Total burden all causes (95% Uncertainty Interval)**	5.4% (5.0-5.6%)	3,305,594	177,032 (166,300-186,100)	2.1% (1.8-2.5%)
**Men**				
Major depressive disorder	23.2%	85,006	19,723	0
Anxiety disorders[&]	38.7%	72,856	28,198	0
PTSD	49.7%	20,435	10,155	0
Other anxiety disorders	34.4%	52,421	18,044	0

Alcohol use disorders	9.9%	44,504	4,399	33
Self-harm[$]	44.4%	114,743	50,997	1,102
Current smoking	14.0%	199,395	27,890	929
**Total burden - mental disorders and health risk behaviours (95% Uncertainty Interval)**	25.4% (23.4-27.0%)	516,503	131,208 (120,700-139,200)	2,064 (1,800-2,300)
**Total burden all causes (95% Uncertainty Interval)**	3.8% (3.5-4.1%)	3,412,485	131,208 (120,700-139,200)	2.2% (1.9-2.5%)

PTSD= post-traumatic stress disorder.

[&]GAD in ACMS is used as a proxy for all other anxiety disorders excluding PTSD; PTSD plus other anxiety disorders attributable DALYs were added to estimate total anxiety disorders attributable burden.

[$]PAFs for suicide attempts (ever) were applied to Australia GBD 2021 burden estimates for intentional self-harm (burden from self-inflicted injuries, including suicides, non-fatal suicide attempts plus self-harm injuries).

Based on simply adjusted model (age, childhood financial stress and geographical remoteness).

Appendix 8 Limitations

Indigenous status

Indigenous status was included in the survey demographics, and as reported elsewhere (Haslam et al. 2023), the sample included representative participation by Indigenous individuals. However, a limitation of our study is that we did not separately analyse outcomes for this group. This decision was made for ethical and methodological reasons. It would not have been ethical to conduct this analysis because our survey was designed as a general population survey and was not designed in accordance with Aboriginal participatory action research principles. In addition, small cell sizes (n=290) precluded meaningful analysis and would have produced unreliable results.

Contribution of multiple risks and mediation

Exposure to child maltreatment often co-occurs within the context of other family dysfunction, social deprivation, and other environmental stressors that are also associated with mental disorders. Although we controlled for other ACEs and bullying victimisation in sensitivity analyses, it is possible that participants have been further exposed to other adversity or other interpersonal violence, such as adult intimate partner violence (IPV), and some of the effects of child maltreatment on mental disorders may be explained by adult victimisation (Messman-Moore et al. 2000, McGuigan & Middlemiss 2005). There may also be important mediators of the effect which are not well understood, where a risk factor may affect another risk factor that lies in the pathway to a disease outcome. For example, high alcohol use is also a risk factor for loss of health, which affects some of the same outcomes (namely self-harm/suicide and alcohol use disorders), and some of the effects of child maltreatment on these outcomes may be mediated through high alcohol use. In our RR estimation, we did not adjust for mediation as our goal was to capture the direct effect of child maltreatment on outcomes.

Appendix 9 List of abbreviations

ABDS=Australian Burden of Disease Study
ACEs=Adverse childhood experiences
ACMS=Australian Child Maltreatment Study
AUD=Alcohol use disorders
BMI=Body mass index
CI=Confidence interval
COVID-19=Coronavirus disease 2019
DALYs=Disability-adjusted life years
EA=Emotional abuse
EDV=Exposure to domestic violence
GAD=Generalised anxiety disorder
ICD-10=International Statistical Classification of Diseases and Related Health Problems, 10th revision
IPV= Intimate Partner Violence
GBD=Global Burden of Disease Study
JVQ=Juvenile Victimization Questionnaire
MINI=Mini International Neuropsychiatric Interview
MDD=Major depressive disorder
NEG=Neglect
NHMRC=National Health and Medical Research Council
OCD=Obsessive-compulsive disorder
PA=Physical abuse
PAF=Population attributable fraction
PTSD=Post-traumatic stress disorder
RR=Relative risk
SA=Sexual abuse
TMREL=Theoretical minimum risk exposure level
UI=Uncertainty interval
YLDs=Years lived with disability
YLLs=Years of life lost

References

Australian Bureau of Statistics (ABS) (2021) Remoteness Structure. Canberra: ABS

Brauer M, Roth GA, Aravkin AY, Zheng P, Abate KH, Abate YH, Abbafati C, Abbasgholizadeh R, Abbasi MA, Abbasian M, Abbasifard M, Abbasi-Kangevari M, Abd ElHafeez S, Abd-Elsalam S, Abdi P, Abdollahi M, Abdoun M, Abdulah DM, Abdullahi A, Abebe M, Abedi A, Abedi A, Abegaz TM, Abeldaño Zuñiga RA, Abiodun O, Abiso TL, Aboagye RG, Abolhassani H, Abouzid M, Aboye GB, Abreu LG, Abualruz H, Abubakar B, Abu-Gharbieh E, Abukhadijah HJJ, Aburuz S, Abu-Zaid A, Adane MM, Addo IY, Addolorato G, Adedoyin RA, Adekanmbi V, Aden B, Adetunji JB, Adeyeoluwa TE, Adha R, Adibi A, Adnani QES, Adzigbli LA, Afolabi AA, Afolabi RF, Afshin A, Afyouni S, Afzal MS, Afzal S, Agampodi SB, Agbozo F, Aghamiri S, Agodi A, Agrawal A, Agyemang-Duah W, Ahinkorah BO, Ahmad A, Ahmad D, Ahmad F, Ahmad N, Ahmad S, Ahmad T, Ahmed A, Ahmed A, Ahmed A, Ahmed LA, Ahmed MB, Ahmed S, Ahmed SA, Ajami M, Akalu GT, Akara EM, Akbarialiabad H, Akhlaghi S, Akinosoglou K, Akinyemiju T, Akkaif MA, Akkala S, Akombi-Inyang B, Al Awaidy S, Al Hasan SM, Alahdab F, AL-Ahdal TMA, Alalalmeh SO, Alalwan TA, Al-Aly Z, Alam K, Alam N, Alanezi FM, Alanzi TM, Albakri A, AlBataineh MT, Aldhaleei WA, Aldridge RW, Alemayohu MA, Alemu YM, Al-Fatly B, Al-Gheethi AAS, Al-Habbal K, Alhabib KF, Alhassan RK, Ali A, Ali A, Ali BA, Ali I, Ali L, Ali MU, Ali R, Ali SSS, Ali W, Alicandro G, Alif SM, Aljunid SM, Alla F, Al-Marwani S, Al-Mekhlafi HM, Almustanyir S, Alomari MA, Alonso J, Alqahtani JS, Alqutaibi AY, Al-Raddadi RM, Alrawashdeh A, Al-Rifai RH, Alrousan SM, Al-Sabah SK, Alshahrani NZ, Altaany Z, Altaf A, Al-Tawfiq JA, Altirkawi KA, Aluh DO, Alvis-Guzman N, Alvis-Zakzuk NJ, Alwafi H, Al-Wardat MS, Al-Worafi YM, Aly H, Aly S, Alzoubi KH, Al-Zyoud W, Amaechi UA, Aman Mohammadi M, Amani R, Amiri S, Amirzade-Iranaq MH, Ammirati E, Amu H, Amugsi DA, Amusa GA, Ancuceanu R, Anderlini D, Anderson JA, Andrade PP, Andrei CL, Andrei T, Anenberg SC, Angappan D, Angus C, Anil A, Anil S, Anjum A, Anoushiravani A, Antonazzo IC, Antony CM, Antriyandarti E, Anuoluwa BS, Anvari D, Anvari S, Anwar S, Anwar SL, Anwer R, Anyabolo EE, Anyasodor AE, Apostol GLC, Arabloo J, Arabzadeh Bahri R, Arafat M, Areda D, Aregawi BB, Aremu A, Armocida B, Arndt MB, Ärnlöv J, Arooj M, Artamonov AA, Artanti KD, Aruleba IT, Arumugam A, Asbeutah AM, Asgary S, Asgedom AA, Ashbaugh C, Ashemo MY, Ashraf T, Askarinejad A, Assmus M, Astell-Burt T, Athar M, Athari SS, Atorkey P, Atreya A, Aujayeb A, Ausloos M, Avila-Burgos L, Awoke AA, Ayala Quintanilla BP, Ayatollahi H, Ayestas Portugal C, Ayuso-Mateos JL, Azadnajafabad S, Azevedo RMS, Azhar GS, Azizi H, Azzam AY, Backhaus IL, Badar M, Badiye AD, Bagga A, Baghdadi S, Bagheri N, Bagherieh S, Bahrami Taghanaki P, Bai R, Baig AA, Baker JL, Bakkannavar SM, Balasubramanian M, Baltatu OC, Bam K, Bandyopadhyay S, Banik B, Banik PC, Banke-Thomas A, Bansal H, Barchitta M, Bardhan M, Bardideh E, Barker-Collo SL, Bärnighausen TW, Barone-Adesi F, Barqawi HJ, Barrero LH, Barrow A, Barteit S, Basharat Z, Basiru A, Basso JD, Bastan M-M, Basu S, Batchu S, Batra K, Batra R, Baune BT, Bayati M, Bayileyegn NS, Beaney T, Behnoush AH, Beiranvand M, Béjot Y, Bekele A, Belgaumi UI, Bell AW, Bell ML, Bello MB, Bello OO, Belo L, Beloukas A, Bendak S, Bennett DA, Bennitt FB, Bensenor IM, Benzian H, Beran A, Berezvai Z, Bernabe E, Bernstein RS, Bettencourt PJG, Bhagavathula AS, Bhala N, Bhandari D, Bhardwaj N, Bhardwaj P, Bhaskar S, Bhat AN, Bhat V, Bhatti GK, Bhatti JS, Bhatti MS, Bhatti R, Bhuiyan MA, Bhutta ZA, Bikbov B, Bishai JD, Bisignano C, Biswas A, Biswas B, Biswas RK, Bjørge T, Boachie MK, Boakye H, Bockarie MJ, Bodolica V, Bodunrin AO, Bogale EK, Bolla SR, Boloor A, Bonakdar Hashemi M, Boppana SH, Bora Basara B, Borhany H, Botero Carvajal A, Bouaoud S, Boufous S, Bourne R, Boxe C, Braithwaite D, Brant LC, Brar A, Breitborde NJK, Breitner S, Brenner H, Briko AN, Britton G, Brown CS, Browne AJ, Brunoni AR, Bryazka D, Bulamu NB, Bulto LN, Buonsenso D, Burkart K, Burns RA, Busse R, Bustanji Y, Butt NS, Butt ZA, Caetano dos Santos FL, Cagney J, Cahuana-Hurtado L, Calina D, Cámera LA, Campos LA, Campos-Nonato IR, Cao C, Cao F, Cao

Y, Capodici A, Cárdenas R, Carr S, Carreras G, Carrero JJ, Carugno A, Carvalho F, Carvalho M, Castaldelli-Maia JM, Castañeda-Orjuela CA, Castelpietra G, Catalá-López F, Catapano AL, Cattaruzza MS, Caye A, Cederroth CR, Cegolon L, Cenderadewi M, Cercy KM, Cerin E, Chadwick J, Chakraborty C, Chakraborty PA, Chakraborty S, Chan JSK, Chan RNC, Chandan JS, Chandika RM, Chaturvedi P, Chen A-T, Chen CS, Chen H, Chen MX, Chen M, Chen S, Cheng C-Y, Cheng ETW, Cherbuin N, Chi G, Chichagi F, Chimed-Ochir O, Chimoriya R, Ching PR, Chirinos-Caceres JL, Chitheer A, Cho WCS, Chong B, Chopra H, Chowdhury R, Christopher DJ, Chu D-T, Chukwu IS, Chung E, Chung S-C, Chutiyami M, Cioffi I, Cogen RM, Cohen AJ, Columbus A, Conde J, Corlateanu A, Cortese S, Cortesi PA, Costa VM, Costanzo S, Criqui MH, Cruz JA, Cruz-Martins N, Culbreth GT, da Silva AG, Dadras O, Dai X, Dai Z, Daikwo PU, Dalli LL, Damiani G, D'Amico E, D'Anna L, Darwesh AM, Das JK, Das S, Dash NR, Dashti M, Dávila-Cervantes CA, Davis Weaver N, Davitoiu DV, De la Hoz FP, de la Torre-Luque A, De Leo D, Debopadhaya S, Degenhardt L, Del Bo' C, Delgado-Enciso I, Delgado-Saborit JM, Demoze CK, Denova-Gutiérrez E, Dervenis N, Dervišević E, Desai HD, Desai R, Devanbu VGC, Dewan SMR, Dhali A, Dhama K, Dhane AS, Dhimal ML, Dhimal M, Dhingra S, Dhulipala VR, Dhungana RR, Dias da Silva D, Diaz D, Diaz LA, Diaz MJ, Dima A, Ding DD, Dinu M, Djalalinia S, Do TC, Do THP, do Prado CB, Dodangeh M, Dohare S, Dokova KG, Dong W, Dongarwar D, D'Oria M, Dorostkar F, Dorsey ER, Doshi R, Doshmangir L, Dowou RK, Driscoll TR, Dsouza AC, Dsouza HL, Dumith SC, Duncan BB, Duraes AR, Duraisamy S, Dushpanova A, Dzianach PA, Dziedzic AM, Ebrahimi A, Echieh CP, Ed-Dra A, Edinur HA, Edvardsson D, Edvardsson K, Efendi F, Eftekharimehrabad A, Eini E, Ekholuenetale M, Ekundayo TC, El Arab RA, El Sayed Zaki M, El-Dahiyat F, Elemam NM, Elgar FJ, ElGohary GMT, Elhabashy HR, Elhadi M, Elmehrath AO, Elmeligy OAA, Elshaer M, Elsohaby I, Emeto TI, Esfandiari N, Eshrati B, Eslami M, Esmaeili SV, Estep K, Etaee F, Fabin N, Fagbamigbe AF, Fagbule OF, Fahimi S, Falzone L, Fareed M, Farinha CS e S, Faris MEM, Faris PS, Faro A, Fasina FO, Fatehizadeh A, Fauk NK, Fazylov T, Feigin VL, Feng X, Fereshtehnejad S-M, Feroze AH, Ferrara P, Ferrari AJ, Ferreira N, Fetensa G, Feyisa BR, Filip I, Fischer F, Fitriana I, Flavel J, Flohr C, Flood D, Flor LS, Foigt NA, Folayan MO, Force LM, Fortuna D, Foschi M, Franklin RC, Freitas A, Friedman SD, Fux B, G S, Gaal PA, Gaihre S, Gajdács M, Galali Y, Gallus S, Gandhi AP, Ganesan B, Ganiyani MA, Garcia V, Gardner WM, Garg RK, Gautam RK, Gebi TG, Gebregergis MW, Gebrehiwot M, Gebremariam TBB, Gebremeskel TG, Gerema U, Getacher L, Getahun GK a, Getie M, Ghadirian F, Ghafarian S, Ghaffari Jolfayi A, Ghailan KY, Ghajar A, Ghasemi M, Ghasempour Dabaghi G, Ghasemzadeh A, Ghassemi F, Ghazy RM, Gholami A, Gholamrezanezhad A, Gholizadeh N, Ghorbani M, Gil AU, Gil GF, Gilbertson NM, Gill PS, Gill TK, Gindaba EZ, Girmay A, Glasbey JC, Gnedovskaya EV, Göbölös L, Godinho MA, Goel A, Golechha M, Goleij P, Golinelli D, Gomes NGM, Gopalani SV, Gorini G, Goudarzi H, Goulart AC, Gouravani M, Goyal A, Graham SM, Grivna M, Grosso G, Guan S-Y, Guarducci G, Gubari MIM, Guha A, Guicciardi S, Gulati S, Gulisashvili D, Gunawardane DA, Guo C, Gupta AK, Gupta B, Gupta M, Gupta R, Gupta RD, Gupta R, Gupta S, Gupta VB, Gupta VK, Gupta VK, Habibzadeh F, Habibzadeh P, Hadaro TS, Hadian Z, Haep N, Haghi-Aminjan H, Haghmorad D, Hagins H, Haile D, Hailu A, Hajj Ali A, Halboub ES, Halimi A, Hall BJ, Haller S, Halwani R, Hamadeh RR, Hamdy NM, Hameed S, Hamidi S, Hammoud A, Hanif A, Hanifi N, Haq ZA, Haque MR, Harapan H, Hargono A, Haro JM, Hasaballah AI, Hasan I, Hasan MJ, Hasan SMM, Hasani H, Hasanian M, Hashmeh N, Hasnain MS, Hassan A, Hassan I, Hassan Zadeh Tabatabaei MS, Hassani S, Hassanipour S, Hassankhani H, Haubold J, Havmoeller RJ, Hay SI, Hebert JJ, Hegazi OE, Hegena TY, Heidari G, Heidari M, Helfer B, Hemmati M, Henson CA, Herbert ME, Herteliu C, Heuer A, Hezam K, Hinneh TK, Hiraike Y, Hoan NQ, Holla R, Hon J, Hoque ME, Horita N, Hossain S, Hosseini SE, Hosseinzadeh H, Hosseinzadeh M, Hostiuc M, Hostiuc S, Hoven H, Hsairi M, Hsu JM, Hu C, Huang J, Huda MN, Hulland EN, Hultström M, Hushmandi K, Hussain J, Hussein NR, Huynh

CK, Huynh H-H, Ibitoye SE, Idowu OO, Ihler AL, Ikeda N, Ikuta KS, Ilesanmi OS, Ilic IM, Ilic MD, Imam MT, Immurana M, Inbaraj LR, Irham LM, Isa MA, Islam MR, Ismail F, Ismail NE, Iso H, Isola G, Iwagami M, Iwu CCD, Iwu-Jaja CJ, J V, Jaafari J, Jacob L, Jacobsen KH, Jadidi-Niaragh F, Jahankhani K, Jahanmehr N, Jahrami H, Jain A, Jain N, Jairoun AA, Jaiswal A, Jakovljevic M, Jalilzadeh Yengejeh R, Jamora RDG, Jatau AI, Javadov S, Javaheri T, Jayaram S, Jeganathan J, Jeswani BM, Jiang H, Johnson CO, Jokar M, Jomehzadeh N, Jonas JB, Joo T, Joseph A, Joseph N, Joshi V, Joshua CE, Jozwiak JJ, Jürisson M, Kaambwa B, Kabir A, Kabir Z, Kadashetti V, Kahn EM, Kalani R, Kaliyadan F, Kalra S, Kamath R, Kanagasabai T, Kanchan T, Kandel H, Kanmiki EW, Kanmodi KK, Kansal SK, Kapner DJ, Kapoor N, Karagiannidis E, Karajizadeh M, Karakasis P, Karanth SD, Karaye IM, Karch A, Karim A, Karimi H, Karmakar S, Kashoo FZ, Kasraei H, Kassahun WD, Kassebaum NJ, Kassel MB, Katikireddi SV, Kauppila JH, Kawakami N, Kaydi N, Kayode GA, Kazemi F, Keiyoro PN, Kemmer L, Kempen JH, Kerr JA, Kesse-Guyot E, Khader YS, Khafaie MA, Khajuria H, Khalaji A, Khalil M, Khalilian A, Khamesipour F, Khan A, Khan MN, Khan M, Khan MJ, Khan MA, Khanmohammadi S, Khatab K, Khatatbeh H, Khatatbeh MM, Khatib MN, Khavandegar A, Khayat Kashani HR, Khidri FF, Khodadoust E, Khormali M, Khorrami Z, Khosla AA, Khosrowjerdi M, Khreis H, Khusun H, Kifle ZD, Kim K, Kim MS, Kim YJ, Kimokoti RW, Kisa A, Kisa S, Knibbs LD, Knudsen AKS, Koh DSQ, Kolahi A-A, Kompani F, Kong J, Koren G, Korja M, Korshunov VA, Korzh O, Kosen S, Kothari N, Koul PA, Koulmane Laxminarayana SL, Krishan K, Krishnamoorthy V, Krishnamoorthy Y, Krishnan B, Krohn KJ, Kuate Defo B, Kucuk Bicer B, Kuddus MA, Kuddus M, Kugbey N, Kuitunen I, Kulimbet M, Kulkarni V, Kumar A, Kumar N, Kumar V, Kundu S, Kurmi OP, Kusnali A, Kusuma D, Kutluk T, La Vecchia C, Ladan MA, Laflamme L, Lahariya C, Lai DTC, Lal DK, Lallukka T, Lám J, Lan Q, Lan T, Landires I, Lanfranchi F, Langguth B, Lansingh VC, Laplante-Lévesque A, Larijani B, Larsson AO, Lasrado S, Lauriola P, Le H-H, Le LKD, Le NHH, Le TTT, Leasher JL, Ledda C, Lee M, Lee PH, Lee SW, Lee SWH, Lee YH, LeGrand KE, Leigh J, Leong E, Lerango TL, Lescinsky H, Leung J, Li M-C, Li W-Z, Li W, Li Y, Li Z, Ligade VS, Lim L-L, Lim SS, Lin R-T, Lin S, Liu C, Liu G, Liu J, Liu J, Liu RT, Liu S, Liu W, Liu X, Liu X, Livingstone KM, Llanaj E, Lohiya A, López-Bueno R, Lopukhov PD, Lorkowski S, Lotufo PA, Lozano R, Lubinda J, Lucchetti G, Luo L, lv H, M Amin HI, Ma ZF, Maass KL, Mabrok M, Machairas N, Machoy M, Mafhoumi A, Magdy Abd El Razek M, Maghazachi AA, Mahadeshwara Prasad DR, Maharaj SB, Mahmoud MA, Mahmoudi E, Majeed A, Makram OM, Makris KC, Malasala S, Maled V, Malhotra K, Malik AA, Malik I, Malinga LA, Malta DC, Mamun AA, Manda AL, Manla Y, Mansour A, Mansouri B, Mansouri P, Mansourian M, Mansournia MA, Mantovani LG, Manu E, Marateb HR, Maravilla JC, Marsh E, Martinez G, Martinez-Piedra R, Martini S, Martins-Melo FR, Martorell M, Marx W, Maryam S, Mathangasinghe Y, Mathioudakis AG, Matozinhos FP, Mattumpuram J, Maugeri A, Maulik PK, Mayeli M, Mazidi M, Mazzotti A, McGrath JJ, McKee M, McKowen ALW, McLaughlin SA, McPhail MA, McPhail SM, Mechili EA, Mehmood A, Mehmood K, Mehrabani-Zeinabad K, Mehrabi Nasab E, Meier T, Mejia-Rodriguez F, Mekene Meto T, Mekonnen BD, Menezes RG, Mengist B, Mensah GA, Mensah LG, Mentis A-FA, Meo SA, Meretoja A, Meretoja TJ, Mersha AM, Mesfin BA, Mestrovic T, Mettananda KCD, Mettananda S, Miazgowski T, Micha G, Michalek IM, Micheletti Gomide Nogueira de Sá AC, Miller TR, Mirarefin M, Mirghafourvand M, Mirica A, Mirijello A, Mirrakhimov EM, Mirshahi A, Mirzaei M, Mishra AK, Mishra V, Mitchell PB, Mithra P, Mittal C, Moazen B, Moberg ME, Mocciaro G, Mohamadkhani A, Mohamed AZ, Mohamed AI, Mohamed J, Mohamed MFH, Mohamed NS, Mohammadi E, Mohammadi S, Mohammadian-Hafshejani A, Mohammadifard N, Mohammed H, Mohammed M, Mohammed S, Mohammed S, Mokdad AH, Monasta L, Mondello S, Moni MA, Moodi Ghalibaf A, Moore CE, Moradi M, Moradi Y, Moraga P, Morawska L, Moreira RS, Morovatdar N, Morrison SD, Morze J, Mosaddeghi Heris R, Mossialos E, Motappa R, Mougin V, Mousavi P, Msherghi A, Mubarik S, Muccioli L, Mueller UO,

Mulita F, Mullany EC, Munjal K, Murillo-Zamora E, Murlimanju B, Musina A-M, Mustafa G, Muthu S, Muthupandian S, Muthusamy R, Muzaffar M, Myung W, Nafei A, Nagarajan AJ, Nagaraju SP, Nagel G, Naghavi M, Naghavi P, Naik GR, Naik G, Nainu F, Nair TS, Najdaghi S, Nakhostin Ansari N, Nanavaty DP, Nangia V, Narasimha Swamy S, Narimani Davani D, Nascimento BR, Nascimento GG, Nashwan AJ, Natto ZS, Nauman J, Navaratna SNK, Naveed M, Nayak BP, Nayak VC, Ndejjo R, Nduaguba SO, Negash H, Negoi I, Negoi RI, Nejadghaderi SA, Nejjari C, Nematollahi MH, Nepal S, Neupane S, Ng M, Nguefack-Tsague G, Ngunjiri JW, Nguyen DH, Nguyen NNY, Nguyen PT, Nguyen PT, Nguyen VT, Nguyen Tran Minh D, Niazi RK, Nicholson SI, Nie J, Nikoobar A, Nikpoor AR, Ningrum DNA, Nnaji CA, Noman EA, Nomura S, Noroozi N, Norrving B, Noubiap JJ, Nri-Ezedi CA, Ntaios G, Ntsekhe M, Nunemo MH, Nurrika D, Nutor JJ, Oancea B, O'Connell EM, Odetokun IA, O'Donnell MJ, Oduro MS, Ogunfowokan AA, Ogunkoya A, Oh I-H, Okati-Aliabad H, Okeke SR, Okekunle AP, Okonji OC, Olagunju AT, Olasupo OO, Olatubi MI, Oliveira AB, Oliveira GMM, Olorukooba AA, Olufadewa II, Olusanya BO, Olusanya JO, Oluwafemi YD, Omar HA, Omar Bali A, Omer GL, Ong KL, Ong S, Onwujekwe OE, Onyedibe KI, Oppong AF, Ordak M, Orish VN, Ornello R, Orpana HM, Ortiz A, Ortiz-Prado E, Osman WMS, Ostroff SM, Osuagwu UL, Otoiu A, Otstavnov N, Otstavnov SS, Ouyahia A, Owolabi MO, Oyeyemi IT, Oyeyemi OT, P A MP, Pacheco-Barrios K, Padron-Monedero A, Padubidri JR, Pal PK, Palicz T, Pan F, Pan H-F, Pana A, Panda SK, Panda-Jonas S, Pandey A, Pandi-Perumal SR, Pangaribuan HU, Pantazopoulos I, Pantea Stoian AM, Papadopoulou P, Parent MC, Parija PP, Parikh RR, Park S, Park S, Parsons N, Pashaei A, Pasovic M, Passera R, Patil S, Patoulias D, Patthipati VS, Paudel U, Pawar S, Pazoki Toroudi H, Peden AE, Pedersini P, Peng M, Pensato U, Pepito VCF, Peprah EK, Peprah P, Peres MFP, Perianayagam A, Perico N, Perna S, Pesudovs K, Petcu I-R, Petermann-Rocha FE, Pham HT, Philip AK, Phillips MR, Pickering BV, Pierannunzio D, Pigeolet M, Pigott DM, Piracha ZZ, Piradov MA, Pisoni E, Piyasena MP, Plass D, Plotnikov E, Poddighe D, Polkinghorne KR, Poluru R, Pond CD, Popovic DS, Porru F, Postma MJ, Poudel GR, Pour-Rashidi A, Pourshams A, Pourtaheri N, Prabhu D, Prada SI, Pradhan J, Pradhan PMS, Prasad M, Prates EJS, Purnobasuki H, Purohit BM, Puvvula J, Qasim NH, Qattea I, Qazi AS, Qian G, Qiu S, Rabiee Rad M, Radfar A, Radhakrishnan RA, Radhakrishnan V, Raeisi Shahraki H, Rafferty Q, Rafiei A, Raggi A, Raghav PR, Raheem N, Rahim F, Rahim MJ, Rahimifard M, Rahimi-Movaghar V, Rahman MO, Rahman MA, Rahmani AM, Rahmani B, Rahmanian M, Rahmanian N, Rahmanian V, Rahmati M, Rahmawaty S, Raimondo D, Rajaa S, Rajendran V, Rajput P, Ramadan MM, Ramasamy SK, Ramasubramani P, Ramazanu S, Ramteke PW, Rana J, Rana K, Ranabhat CL, Rane A, Rani U, Ranta A, Rao CR, Rao M, Rao PC, Rao SJ, Rasella D, Rashedi S, Rashedi V, Rashidi M, Rashidi M-M, Rasouli-Saravani A, Ratan ZA, Rathnaiah Babu G, Rauniyar SK, Rautalin I, Rawaf DL, Rawaf S, Rawassizadeh R, Razo C, Reda ZFF, Reddy MMRK, Redwan EMM, Reifels L, Reitsma MB, Remuzzi G, Reshmi B, Resnikoff S, Restaino S, Reyes LF, Rezaei M, Rezaei N, Rezaei N, Rezaeian M, Rhee TG, Riaz MA, Ribeiro ALP, Rickard J, Robinson-Oden HE, Rodrigues CF, Rodrigues M, Rodriguez JAB, Roever L, Romadlon DS, Ronfani L, Rosauer JJ, Roshandel G, Rostamian M, Rotimi K, Rout HS, Roy B, Roy N, Rubagotti E, Ruela G de A, Rumisha SF, Runghien T, Russo M, Ruzzante SW, S N C, Saad AMA, Saber K, Saber-Ayad MM, Sabour S, Sacco S, Sachdev PS, Sachdeva R, Saddik B, Saddler A, Sadee BA, Sadeghi E, Sadeghi M, Sadeghi Majd E, Saeb MR, Saeed U, Safari M, Safi S, Safi SZ, Sagar R, Sagoe D, Saheb Sharif-Askari F, Saheb Sharif-Askari N, Sahebkar A, Sahoo SS, Sahu M, Saif Z, Sajid MR, Sakshaug JW, Salam N, Salamati P, Salami AA, Salaroli LB, Salehi L, Salehi S, Salem MR, Salem MZY, Salihu D, Salimi S, Salum GA, Samadi Kafil H, Samadzadeh S, Samodra YL, Samuel VP, Samy AM, Sanabria J, Sanjeev RK, Sanna F, Santomauro DF, Santric-Milicevic MM, Sarasmita MA, Saraswathy SYI, Saravanan A, Saravi B, Sarikhani Y, Sarmiento-Suárez R, Sarode GS, Sarode SC, Sartorius B, Sarveazad A, Sathian B, Sattin D, Sawhney M, Saya GK, Sayeed A, Sayeed MA, Sayyah M, Schinckus C, Schmidt MI, Schuermans A,

Schumacher AE, Schutte AE, Schwarzinger M, Schwebel DC, Schwendicke F, Selvaraj S, Semreen MH, Senthilkumaran S, Serban D, Serre ML, Sethi Y, Shafie M, Shah H, Shah NS, Shah PA, Shah SM, Shahbandi A, Shaheen AA, Shahid S, Shahid W, Shahsavari HR, Shahwan MJ, Shaikh MA, Shaikh SZ, Shalash AS, Sham S, Shamim MA, Shams-Beyranvand M, Shamshirgaran MA, Shamsi MA, Shanawaz M, Shankar A, Sharfaei S, Sharifan A, Sharifi-Rad J, Sharma M, Sharma U, Sharma V, Shastry RP, Shavandi A, Shehabeldine AME, Shehzadi S, Sheikh A, Shen J, Shetty A, Shetty BSK, Shetty PH, Shiani A, Shiferaw D, Shigematsu M, Shin M-J, Shiri R, Shittu A, Shiue I, Shivakumar KM, Shivarov V, Shool S, Shorofi SA, Shrestha R, Shrestha S, Shuja KH, Shuval K, Si Y, Siddig EE, Silva DAS, Silva LMLR, Silva S, Silva TPR, Simpson CR, Singh A, Singh BB, Singh B, Singh G, Singh H, Singh JA, Singh M, Singh NP, Singh P, Singh S, Sinto R, Sivakumar S, Siwal SS, Skhvitaridze N, Skou ST, Sleet DA, Sobia F, Soboka M, Socea B, Solaimanian S, Solanki R, Solanki S, Soliman SSM, Somayaji R, Song Y, Sorensen RJD, Soriano JB, Soyiri IN, Spartalis M, Spearman S, Spencer CN, Sreeramareddy CT, Stachteas P, Stafford LK, Stanaway JD, Stanikzai MH, Stein C, Stein DJ, Steinbeis F, Steiner C, Steinke S, Steiropoulos P, Stockfelt L, Stokes MA, Straif K, Stranges S, Subedi N, Subramaniyan V, Suleman M, Suliankatchi Abdulkader R, Sundström J, Sunkersing D, Sunnerhagen KS, Suresh V, Swain CK, Szarpak L, Szeto MD, Tabaee Damavandi P, Tabarés-Seisdedos R, Tabatabaei SM, Tabatabaei Malazy O, Tabatabaeizadeh S-A, Tabatabai S, Tabche C, Tabish M, Tadakamadla SK, Taheri Abkenar Y, Taheri Soodejani M, Taherkhani A, Taiba J, Takahashi K, Talaat IM, Tamuzi JL, Tan K-K, Tang H, Tat NY, Taveira N, Tefera YM, Tehrani-Banihashemi A, Temesgen WA, Temsah M-H, Teramoto M, Terefa DR, Teye-Kwadjo E, Thakur R, Thangaraju P, Thankappan KR, Thapar R, Thayakaran R, Thirunavukkarasu S, Thomas N, Thomas NK, Tian J, Tichopad A, Ticoalu JHV, Tiruye TY, Tobe-Gai R, Tolani MA, Tolossa T, Tonelli M, Topor-Madry R, Topouzis F, Touvier M, Tovani-Palone MR, Trabelsi K, Tran JT, Tran MTN, Tran NM, Trico D, Trihandini I, Troeger CE, Tromans SJ, Truyen TTTT, Tsatsakis A, Tsermpini EE, Tumurkhuu M, Udoakang AJ, Udoh A, Ullah A, Ullah S, Ullah S, Umair M, Umakanthan S, Unim B, Unnikrishnan B, Upadhyay E, Urso D, Usman JS, Vaithinathan AG, Vakili O, Valenti M, Valizadeh R, Van den Eynde J, van Donkelaar A, Varga O, Vart P, Varthya SB, Vasankari TJ, Vasic M, Vaziri S, Venketasubramanian N, Verghese NA, Verma M, Veroux M, Verras G-I, Vervoort D, Villafañe JH, Villalobos-Daniel VE, Villani L, Villanueva GI, Vinayak M, Violante FS, Vlassov V, Vo B, Vollset SE, Volovat SR, Vos T, Vujcic IS, Waheed Y, Wang C, Wang F, Wang S, Wang Y, Wang Y-P, Wanjau MN, Waqas M, Ward P, Waris A, Wassie EG, Weerakoon KG, Weintraub RG, Weiss DJ, Weiss EJ, Weldetinsaa HLL, Wells KM, Wen YF, Wiangkham T, Wickramasinghe ND, Wilkerson C, Willeit P, Wilson S, Wong YJ, Wongsin U, Wozniak S, Wu C, Wu D, Wu F, Wu Z, Xia J, Xiao H, Xu S, Xu X, Xu YY, Yadav MK, Yaghoubi S, Yamagishi K, Yang L, Yano Y, Yaribeygi H, Yasufuku Y, Ye P, Yesodharan R, Yesuf SA, Yezli S, Yi S, Yiğit A, Yigzaw ZA, Yin D, Yip P, Yismaw MB, Yon DK, Yonemoto N, You Y, Younis MZ, Yousefi Z, Yu C, Yu Y, Zadey S, Zadnik V, Zakham F, Zaki N, Zakzuk J, Zamagni G, Zaman SB, Zandieh GGZ, Zanghì A, Zar HJ, Zare I, Zarimeidani F, Zastrozhin MS, Zeng Y, Zhai C, Zhang AL, Zhang H, Zhang L, Zhang M, Zhang Y, Zhang Z, Zhang Z-J, Zhao H, Zhao JT, Zhao X-JG, Zhao Y, Zhao Y, Zhong C, Zhou J, Zhou J, Zhou S, Zhu B, Zhu L, Zhu Z, Ziaeian B, Ziafati M, Zielińska M, Zimsen SRM, Zoghi G, Zoller T, Zumla A, Zyoud SH, Zyoud SH, Murray CJL and Gakidou E (2024) Global burden and strength of evidence for 88 risk factors in 204 countries and 811 subnational locations, 1990–2021: a systematic analysis for the Global Burden of Disease Study 2021. *The Lancet* **403**, 2162–2203

GBD Collaborative Network (2021) Global Burden of Disease Study 2021 (GBD 2021) Results. Seattle: Institute for Health Metrics and Evaluation (IHME). Available from https://vizhub.healthdata.org/gbd-results (accessed 18 January 2025)

Hamby S (2014) Intimate partner and sexual violence research: Scientific progress, scientific challenges, and gender. *Trauma, Violence, & Abuse* **15**, 149–158

Haslam DM, Lawrence DM, Mathews B, Higgins DJ, Hunt A, Scott JG, Dunne MP, Erskine HE, Thomas HJ, Finkelhor D, Pacella R, Meinck F and Malacova E (2023) The Australian Child Maltreatment Study (ACMS), a national survey of the prevalence of child maltreatment and its correlates: methodology. *Medical Journal of Australia* **218**, S5–S12

Mathews B, Meinck F, Erskine HE, Tran N, Lee H, Kellard K, Pacella R, Scott JG, Finkelhor D and Higgins DJ (2023a) Adaptation and validation of the Juvenile Victimization Questionnaire-R2 for a national study of child maltreatment in Australia. *Child Abuse & Neglect* **139**, 106093

Mathews B, Pacella R, Dunne M, Scott J, Finkelhor D, Meinck F, Higgins DJ, Erskine H, Thomas HJ, Haslam D, Tran N, Le H, Honey N, Kellard K and Lawrence D (2021) The Australian Child Maltreatment Study (ACMS): protocol for a national survey of the prevalence of child abuse and neglect, associated mental disorders and physical health problems, and burden of disease. *BMJ Open* **11**, e047074

Mathews B, Pacella R, Dunne MP, Simunovic M and Marston C (2020) Improving measurement of child abuse and neglect: A systematic review and analysis of national prevalence studies. *PLOS ONE* **15**, e0227884

Mathews B, Pacella R, Scott JG, Finkelhor D, Meinck F, Higgins DJ, Erskine HE, Thomas HJ, Lawrence DM, Haslam DM, Malacova E and Dunne MP (2023b) The prevalence of child maltreatment in Australia: findings from a national survey. *Medical Journal of Australia* **218**, S13–S18

McGuigan WM and Middlemiss W (2005) Sexual abuse in childhood and interpersonal violence in adulthood: A cumulative impact on depressive symptoms in women. *Journal of Interpersonal Violence* **20**, 1271–1287

Messman-Moore TL, Long PJ and Siegfried NJ (2000) The revictimization of child sexual abuse survivors: An examination of the adjustment of college women with child sexual abuse, adult sexual assault, and adult physical abuse. *Child maltreatment* **5**, 18–27

Thomas HJ, Mathews BM, Green JG, Haslam DM, Healy KL, Pacella R, Higgins DJ, Finkelhor D, Malacova E, Erskine HE, Lawrence DM, Meinck F and Scott JG (2025) Peer and Sibling Bullying Victimization in Childhood: Prevalence in a National Australian Cohort Aged 16 Years and Older. *International Journal of Bullying Prevention*. https://doi.org/10.1007/s42380-025-00307-3
